# Effects of postnatal corticosteroids on lung development in newborn animals. A systematic review

**DOI:** 10.1038/s41390-024-03114-6

**Published:** 2024-03-16

**Authors:** Irene M. Lok, Kimberley E. Wever, Roos J. S. Vliegenthart, Wes Onland, Anton H. van Kaam, Minke van Tuyl

**Affiliations:** 1 Department of Neonatology, Emma Children’s Hospital Amsterdam UMC, location University of Amsterdam, Meibergdreef 9, Amsterdam, The Netherlands; 2 Amsterdam Reproduction & Development (AR&D) Research Institute, Amsterdam, The Netherlands; 3 Department of Anesthesiology, Pain and Palliative Medicine, Radboud University Medical Center, Nijmegen, The Netherlands; 4 Department of Pediatrics, Leiden University Medical Center, Leiden, The Netherlands

## Abstract

**Background:**

Postnatal systemic corticosteroids reduce the risk of bronchopulmonary dysplasia but the effect depends on timing, dosing, and type of corticosteroids. Animal studies may provide valuable information on these variable effects. This systematic review summarizes the effects of postnatal systemic corticosteroids on lung development in newborn animals.

**Methods:**

A systematic search was performed in PubMed and Embase in December 2022. The protocol was published on PROSPERO (CRD42021177701).

**Results:**

Of the 202 eligible studies, 51 were included. Only newborn rodent studies met the inclusion criteria. Most studies used dexamethasone (98%). There was huge heterogeneity in study outcome measures and corticosteroid treatment regimens. Reporting of study quality indicators was mediocre and risk of bias was unclear due to poor reporting of study methodology. Meta-analysis showed that postnatal corticosteroids caused a decrease in body weight as well as persistent alveolar simplification. Subgroup analyses revealed that healthy animals were most affected.

**Conclusion:**

In newborn rodents, postnatal systemic corticosteroids have a persistent negative effect on body weight and lung development. There was huge heterogeneity in experimental models, mediocre study quality, unclear risk of bias, and very small subgroups for meta-analysis which limited firm conclusions.

**Impact:**

- Postnatal corticosteroids reduce the risk of bronchopulmonary dysplasia but the effect depends on timing, dosing, and type of corticosteroids while the underlying mechanism of this variable effect is unknown.
- This is the first systematic review and meta-analysis of preclinical newborn animal studies reviewing the effect of postnatal systemic corticosteroids on lung development.
- In newborn rodent models, postnatal corticosteroids have a persistent negative effect on body weight and lung alveolarization, especially in healthy animals.

## Background

Postnatal corticosteroids in preterm infants are used to prevent and/or treat developing bronchopulmonary dysplasia (BPD).^1^ BPD is a chronic lung disease and the most common complication of preterm birth.^2^ It is characterized by an arrest in alveolar and pulmonary vascular development and is accompanied by long-term pulmonary and neurological sequelae.^3,4^ Acute and/or chronic inflammation is considered the most important risk factor in the multifactorial etiology of evolving BPD, while chorioamnionitis, oxygen therapy, postnatal sepsis, and mechanical ventilation are the most common causes for this inflammatory response in preterm infants.^5–8^ The rationale for postnatal systemic corticosteroid administration to attenuate the inflammatory process and subsequent development of BPD seems therefore plausible.^9^ Although randomized controlled trials in humans have shown that systemic corticosteroids can reduce BPD, the reported treatment regimens according to (inter)national guidelines are highly variable and the treatment effects seem to be related to patient characteristics and type, timing, and dose of corticosteroids.^10–13^ In addition, studies have shown an increase in neurodevelopmental complications in preterm infants exposed to postnatal corticosteroids, indicating that corticosteroids can have a negative effect on the developing brain and other organs.^9,13–17^ The underlying mechanism for these inconsistent effects of corticosteroids is poorly understood.

Over the last decades, multiple animal studies have been done to investigate the effects of postnatal corticosteroids on lung development and in most of these studies in-depth microscopic analyses of whole lungs were performed, something that is not feasible in human studies. The results of these animal studies might provide insight into underlying mechanisms of why the treatment effects of corticosteroids are so variable in preterm infants. In order to optimize the interpretation of these studies, a systematic review is urgently needed.^18^ Therefore, the aim of this systematic review and meta-analysis was to identify, appraise, and summarize all current literature on the effects of the different types and regimens of postnatal systemic corticosteroids on lung development in healthy and diseased newborn animal models.

## Methods

### Protocol and registration

The review methodology for this work was pre-specified in a protocol registered at PROSPERO (CRD42021177701). The following amendments to the review protocol were made: (1) inclusion of research using *systemic* corticosteroids only; (2) we adjusted our method for duplicate outcome data extraction because of limited resources (see below). This review is reported according to PRISMA guidelines (see Supplement 1 for the completed PRISMA checklist).^19^

### Search strategy and study selection process

A comprehensive search was performed in PubMed and Embase (via Ovid) to identify all published animal studies investigating the effects of postnatal corticosteroids on lung development. Databases were searched for published articles from inception until December 27th, 2022. The search strategy included the components “animal”, “corticosteroids”, “lung”, and “newborn animal”. The full search strings can be found in Supplement 2. Articles were included if the study was an original full-length paper reporting unique outcome data on lung development in newborn mammals receiving postnatal systemic corticosteroids before complete alveolarization. Studies were excluded when: (1) publication types were other than a full-length research article (e.g., reviews and conference abstracts), (2) the study was not performed in mammals, (3) no postnatal corticosteroids were administered, (4) there were no outcomes related to lung development or lung injury reported, or animals were terminated within 24 h after treatment, (5) corticosteroids were administered after complete alveolarization, (6) a non-prematurity related lung disease model was used (e.g., persistent pulmonary hypertension of the neonate, congenital diaphragmatic hernia or meconium aspiration syndrome), (7) there was an unsuitable co-intervention or co-morbidity (e.g., artificial placenta), or (8) the full text was unavailable. All reference lists of retrieved articles were searched for additional studies, which did not lead to any new articles. No language restriction was applied. Inclusion of eligible articles was done in two phases in Rayyan (https://www.rayyan.ai): first screening for eligibility based on title and abstract and a second screening for final inclusion based on the full text. In both phases, two reviewers (IL and RV) independently performed the study selection. In case of discrepancies, a third reviewer (MvT) was consulted.

### Study characteristics and outcome data extraction

We extracted the following characteristics from the included studies: animal species and strain, age at the start of the experiment and at the start of corticosteroid treatment, sex, model for lung injury (if applicable), type of corticosteroid, duration of treatment, route of administration, frequency, dose, age of termination, and co-interventions (if applicable). Study characteristics were extracted by one reviewer (IL).

We extracted the mean, standard deviation, and sample size (*n*) for the control and treated groups for the following outcome measures: all-cause mortality, body length and -weight, lung volume, -weight, -morphometry, -inflammation, -function, -proliferation, -matrix, and vascular morphometry. Outcome data displayed in figures were extracted using ImageJ if no numerical data were available.^20^

### Risk of bias and quality assessment

We assessed the reporting quality of five key quality indicators (“yes” versus “no”), namely any randomization, any blinding, a sample size or power calculation, a conflict of interest statement, and any apparent experimental unit of analysis error (e.g., assigning treatment to a litter, while using individual pups as the unit of analysis). Risk of bias was assessed using SYRCLE’s risk of bias tool for animal studies.^21^ Risk of bias for each bias domain was classified as “low”, “high”, or “unclear”. To be classified as low risk of bias for baseline characteristics, the supplier and strain of the animal, (ratio of) sex, and weight at the start of the experiment had to be specified. Both assessments were performed independently by two reviewers (IL and MvT), with consultation of a third reviewer (KW) in case of discrepancies. The assessors were not blinded to the names of the authors during this process.

### Data synthesis and statistical analyses

Meta-analyses were performed using Review Manager 5.4 (The Cochrane Collaboration, Copenhagen, Denmark) on all outcomes reported in a minimum of five studies. All meta-analyses were performed by computing the standardized difference in means (SMD) with the corresponding 95% confidence intervals (CI) to account for the differences between species and different units of measurement. Data were pooled using a random effects model in all analyses, accounting for anticipated heterogeneity. Heterogeneity was assessed and reported as the *I*^2^ statistic. Subgroup analyses were performed post hoc and a minimum of three studies per subgroup was required. The subgroup injury included animals that were exposed to hyperoxia, lipopolysaccharides (LPS), or bleomycin. Exposure to retinoic acid was not injurious hence those animals were categorized as healthy. To differentiate between acute and chronic effects of corticosteroids on lung development, subgroup analysis based on age at evaluation of these effects was performed. Peak alveolarization was taken as the cut-off for this subgroup, which is around postnatal day 15 in small rodents.^22,23^ For outcomes that did not meet the threshold for meta-analysis a descriptive synthesis was performed. We aimed to assess publication bias using visual inspection of funnel plots for all analyses containing ≥20 studies; however, for none of the outcomes this threshold was reached. No sensitivity analyses were performed.

## Results

### Study selection

A total of 10,409 articles were retrieved by the search on December 27th, 2022. After deduplication, a total of 3909 unique articles were screened based on title and abstract. The majority (*n* = 132) of the 202 articles eligible for full-text screening were excluded due to the administration of corticosteroids after complete alveolarization. A total of 51 studies were included in this review, which were all in English and published between 1985 and 2022 (Supplement 3).^24–74^

### Study characteristics

Study characteristics and outcome measures are described in Table 1. Although the search included all newborn animals, only rodent studies met the inclusion criteria; 39 studies used rats (76%), 11 used mice (22%), and one used guinea pigs. We found substantial differences in study design regarding the start of corticosteroid treatment, route of administration, duration, and frequency of treatment, and length of follow-up. Some studies compared multiple time points at which treatment was initiated, multiple durations of treatment, and/or multiple doses. As a result, a total of 87 treatment protocols were identified. Dexamethasone was the most frequently used corticosteroid (98%).

**Table 1:** Study characteristics

Publication	Start of GC treatment (PND)	Duration of GC treatment (days)	Type of GC	Frequency of GC administration	Route of GC administration	Dose of GC (cumulative)	Lung injury model or co-intervention	Age at evaluation of outcome measure (PND)	Outcome measures
Rat									
Bartolome^24^	6, 10	1	Dexa	Single	s.c	10 mcg/g	None	7, 11	P
Blanco^25^	4	10	Dexa	Daily	s.c	2.5 mcg	None	14, 60	L, LV, Morp
Blanco^26^	18	10	Dexa	Daily	s.c	0.02 mcg/g	Hyperoxia	28	Ma, Morp
Corroyer^27^	1	4	Dexa	Daily tapered	s.c	0.185 mcg/g	None	4, 10, 16, 19, 21, 36	P
Dallas^28^	0, 2, 4, 6	1–14	Dexa	Daily	i.p.	3.0–6.5 mcg/g	Hyperoxia	7, 10, 14	I, L, Mort
Fayon^29^	4	10	Dexa HCS	Daily	s.c	Dexa: 4.4 mcg HCS: 11 mcg/g	None	14	L, LV, Morp, Mort
Floros^30^	5, 14–17	1	Dexa	Single, dose response	i.p.	0.002–20 mcg/g	None	6, 15–18	Morp (SP-A)
Garber^31^	1	14	Dexa	Daily	s.c	1.4 mcg	RA	5, 10, 15, 30, 37, 52	F, L, LV, Morp
Gesche^32^	1, 5, 13, 19	2	BMS	Daily	i.p.	2 mcg/g	rhKGF	3, 7, 15, 21	L, Morp, P
Hu^33^	NR	NR	Dexa	Every other day	i.v.	NR, max 7 mcg/g	Hyperoxia	13	I, L, Mort
Ishikawa^34^	0	14	Dexa	Daily	i.p.	1.3 mcg/g	Bleomycin	14	I, L, Morp, Mort
Kim^35^	5	3	Dexa	Daily tapered	i.p.	0.9 mcg/g	Prenatal BMS, Hyperoxia	8, 14	I, L, Morp, Mort, P, V
Le Cras^36^	3	11	Dexa	Daily	s.c.	2.75 mcg	Hypoxia	14, 70	L, LW, Morp, V
Lee^37^	1	6	Dexa HCS	Daily tapered	i.p.	Dexa: 1.75 mcg/g HCS: 7 mcg/g	Prenatal LPS, Hyperoxia	7, 14	I, L, Morp, Mort
Lindsay^38^	0	1	Dexa	Single	NR	100 mcg/g	Hyperoxia	2	I
Liu^39^	1	3	Dexa	Daily	s.c.	75 mcg	None	3, 5, 7, 10, 14, 21	Morp, P
Luyet^40^	1	4	Dexa	Daily tapered	s.c.	0.185 mcg/g	None	4, 10, 16, 19, 21, 28, 36	P
Massaro^41^	4	10	Dexa	Daily	s.c.	1 mcg	None	9, 14, 20, 28, 60	F, L, LV, Morp, P
Massaro^42^	1	6	Dexa	Daily	s.c.	1 mcg	None	7	L, LW, Morp, Mort, P
Massaro^43^	4	10	Dexa	Daily	s.c.	1 mcg	None	6, 8, 11, 14	L, LV, LW, Ma, Morp, P
Massaro^44^	4	10	Dexa	Daily	s.c.	2.5 mcg	RA	14	L, LV, Morp
Massaro^45^	4	10	Dexa	Daily	s.c.	2.5 mcg	RA	37	L, LV, Morp
Ogasawara^46^	0, 2, 4	1	Dexa	Single	i.m.	1 mcg/g	None	1, 3, 5	I, L, LW, Morp
Özer Bekmez^47^	15	7	Dexa HCS MPS	Daily tapered	i.p.	Dexa: 0.725 mcg/g HCS: 18.1 mg/g MPS: 3.81 mcg/g	Hyperoxia	22	I, L, Morp, Mort, P
Ross^48^	5	3	Dexa	Daily	s.c.	0.75 mcg	RA	14	L, Morp
Roth-Kleiner^49^	1	4	Dexa	Daily tapered	s.c.	0.185 mcg/g	None	4, 10, 21, 60	Morp, V
Roth-Kleiner^50^	1	4	Dexa	Daily tapered	s.c.	0.185 mcg/g	None	3, 4, 6, 10, 16, 21, 36, 60	Ma, Morp
Sahebjami^51^	4	10	Dexa	Daily	s.c.	1 mcg	None	99	F, L, LV, LW, Morp, Mort, P
Schwyter^52^	1	4	Dexa	Daily tapered	s.c.	0.185 mcg/g	None	4, 10, 21, 36, 60	Morp
Shimizu^53^	0, 2, 4	1	Dexa	Single	i.p.	0.2 mcg/g	None	1, 3, 5	Morp
Srinivasan^54^	3, 4	10	Dexa	Daily	s.c.	2.5 mcg	RA	30–39	F, L
Theogaraj^55^	1	7	Dexa	Continuously	via breastmilk	1 mcg/ml in drinking water dams	None	60–80	I, L, LW, V
Thibeault^56^	0	8	Dexa	Daily tapered	s.c.	1.8 mcg/g	Hyperoxia	60	F, L, LV, LW, Mort, Morp, P, V
Tsai^57^	2	4	Dexa	Daily	i.p.	4 mcg/g	None	7, 14, 21	I
Tschanz^58^	2	14	Dexa	Daily	s.c.	1.4 mcg	None	4, 7, 10, 13, 21, 36, 44, 60	L, LV, Morp, V
Tschanz^59^	1	4	Dexa	Daily tapered	s.c.	0.185 mcg/g	None	4, 10, 21, 36, 60	L, LV, Morp, V
Valencia^60^	5	3	Dexa	Daily	i.m.	0.3 or 1.5 mcg/g	None	14, 21, 45	L, LW, Ma, Mort
Veness-Meehan^61^	3	10	Dexa	Daily	NR	2.5 mcg	Hyperoxia, RA	14	L, LV, Ma, Morp, Mort
Zhang^62^	1	14	Dexa	Daily	s.c.	1.4 mcg	RA	5, 10, 15	Morp, P, V
Mouse									
Bhatt^63^	6	4	Dexa	Daily	i.p.	0.4 or 4 or 20 mcg/g	None	10	L, LW, V
Clerch^64^	4	11	Dexa	Daily	s.c	5.5 or 11 mcg	RA	15, 37	Ma, Morp, P, V
Hirooka^65^	3	10	Dexa	Daily with 2 non-injection days	i.p.	4 mcg	RA	38, 90, 200	Morp
Kamei^66^	3	10	Dexa	Daily with 2 non-injection days	s.c.	4 mcg	None	5, 7, 14, 21, 42	L, LW, Ma, Morp
Maden^67^	4	10	Dexa	Daily	s.c.	4 mcg	RA	12 weeks	L, LV, Morp
McGowan^68^	1	7, 11	Dexa	Daily tapered	s.c.	0.4 or 0.6 mcg/g	None	8, 12	Morp
Mi^69^	7	7	Dexa	Daily	i.p.	17.5 mcg/g	Prenatal LPS	14	I, Morp
Miyajima^70^	3	10	Dexa	Daily with 2 non-injection days	s.c.	4 mcg	RA	90	Morp
Ohtsu^71^	14	4	Dexa	Daily	s.c.	0.4 or 4 or 20 mcg/g	Hyperoxia	18	F, Morp, Mort
Stinchcombe^72^	4	10	Dexa	Daily with 2 non-injection days	s.c.	4 mcg	RA, 2 different mouse strains	90	L, LV, Morp
Zhuang^73^	3	7, 11	Dexa	Daily	s.c.	7 or 11 or 2.75 mcg	HO-1 knockout	10, 14	Morp, V
Guinea pig									
Town^74^	0	3	Dexa	Daily	s.c.	30 mcg/g	Prematurity, Hyperoxia	3, 5, 7	I, L, LW, Mort

*GC* glucocorticoid, *PND* postnatal day, *NR* not registered, *Dexa* dexamethasone, *HCS* hydrocortisone, *BMS* betamethasone, *MPS* methylprednisolone, *s.c.* subcutaneous, *i.p.* intraperitoneal, *i.v.* intravenous, *i.m.* intramuscular, *RA* retinoic acid, *rhKGF* recombinant human keratinocyte growth factor, *LPS* lipopolysaccharide, *HO-1* heme oxygenase-1, *P* proliferation, *L* body length or -weight, *LV* lung volume, *Morp* lung morphometry, *Ma* lung matrix, *I* lung inflammation, *Mort* mortality, *SP-A* surfactant protein A, *F* lung function, *V* pulmonary vascular morphometry, *LW* lung weight.

In the majority of protocols, corticosteroids were administered subcutaneously (65%), followed by intraperitoneal injection (24%). Two studies did not report the route of administration.^38,61^ Six studies started corticosteroids at multiple time points, resulting in a total of 64 different age cohorts. In 73% of cohorts, corticosteroids were started in the first 4 days of life, and 86% of protocols started within the first week of life. One study did not report the age at which corticosteroids were started.^33^

The studies that used a weight-based cumulative dose administered a range from 0.02 µg/g to 100 µg/g. In forty-five protocols a standard cumulative dose ranging from 0.4 µg to 75 µg was used. The total number of doses varied between a single dose (35% of studies) up to 14 doses. Twelve studies (24%, nineteen protocols) used tapered doses of corticosteroids. In one study, corticosteroids were administered via breastmilk, which resulted in a plasma concentration of ~15 ng/ml in the newborn animals.^55^

Fifteen studies (31%) used a lung injury model to study the effects of corticosteroids. Ten studies used hyperoxia, while three studies used either hypoxia, intraperitoneal bleomycin, or intra-amniotic LPS.^34,36,37^ One study used a double hit model combining prenatal LPS and hyperoxia, whereas another study in guinea pigs combined prematurity with hyperoxia.^37,74^ All other studies made use of healthy newborn animals. Twelve studies (24%) used retinoic acid as a co-intervention.

### Reporting of study quality indicators

**Fig. 1:**
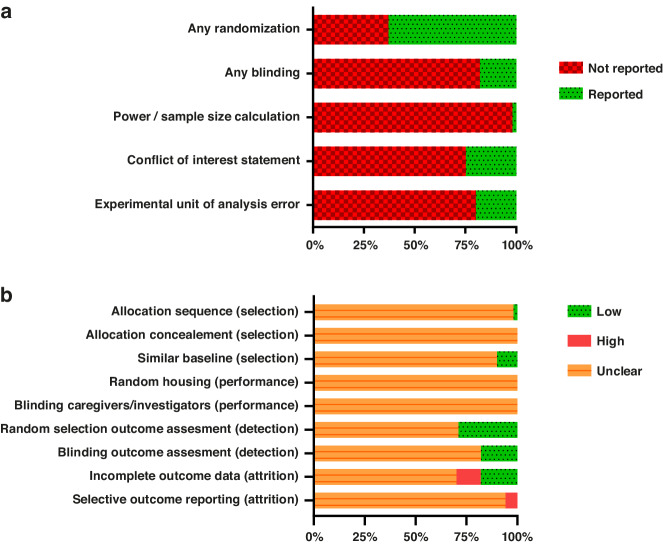
Study quality and risk of bias. Key indicators of study quality (**a**) and risk of bias assessment (**b**) using SYRCLE’s risk of bias tool for animal studies.^21^ The type of bias assessed with each signaling question is indicated between brackets.

The overall reporting of key study quality indicators was mediocre (Fig. 1a). Of the 51 included studies, 32 (63%) mentioned the term randomization at any stage of the experiment and nine studies (18%) reported blinding during any phase of the experiment. In most cases, only the outcome *histology assessment* was blinded. Only one study reported a power calculation to justify the sample size.^47^ A conflict of interest statement was reported in thirteen studies (25%). Forty-one studies (80%) had an error in the experimental unit of analysis, most often because treatment was assigned per litter or not reported, while in the analysis the individual pups were used as the experimental unit.

### Risk of bias

In most studies, multiple risks of bias domains were assessed as unclear (Fig. 1b) due to poor reporting of the study methodology. Five studies (10%) had groups with similar characteristics at baseline, while all other studies were assessed as unclear. Fifteen studies (29%) used some form of random selection during outcome assessment, there was some form of blinding in nine studies (18%), and the risk of attrition bias was high in six studies (12%). Three studies (8%) were assessed as high risk for selective outcome reporting.^30,59,71^ Furthermore, two studies were assessed as high risk for other bias.^55,71^

### Outcome assessment

A description of the outcome mortality can be found in Supplement 4. We were unable to identify five or more studies reporting on similar outcome measures for lung inflammation, -function, -proliferation, -matrix, or vascular morphometry. Therefore, no meta-analysis could be performed for these outcomes and because of heterogeneity between these studies, no pattern in these outcomes could be found.

### Meta-analyses

All meta-analyses and subgroup analyses can be found in Table 2.

**Table 2:** Meta- and subgroup analyses for the outcomes body weight (growth), lung volume and -weight, number of alveoli (RAC), alveolar mean chord length (*L*_m_), wall thickness, airspace volume, and surface area

Outcome measures and subgroups	Studies (*N*)	Comparisons (*N*)	Animals (*N*)	Control (*N*)	Corticosteroid (*N*)	Comparisons				Subgroup difference		
						SMD	95% CI	*I*^2^	*P* value	Chi^2^	*I*^2^	*P* value
Body weight	22	88	1487	697	790	−1.72	−2.08, −1.35	85	<0.01			
<PND 15	16	60	1025	485	540	−2.35	−2.82, −1.87	84	<0.01	34.60	97.1	<0.01
≥PND 15	12	28	462	212	250	−0.51	−0.90, −0.12	69	0.01			
Healthy	19	75	1230	590	640	−1.83	−2.27, −1.40	86	<0.01	1.86	46.1	0.17
Injury	5	13	257	107	150	−1.33	−1.91, −0.74	73	<0.01			
Lung volume	9	35	357	167	190	0.32	−0.03, 0.66	50	0.07			
<PND 15	7	19	194	86	108	0.09	−0.31, 0.49	35	0.65	2.31	56.7	0.13
≥PND 15	6	16	163	81	82	0.64	0.05, 1.23	60	0.03			
Lung weight	6	12	155	61	94	−0.21	−0.63, 0.21	31	0.33			
RAC	11	35	513	242	271	−2.04	−2.93, −1.16	90	<0.01			
<PND 15	8	20	325	162	163	−2.02	−3.18, −0.86	91	<0.01	0.01	0	0.92
≥PND 15	6	15	188	80	108	−2.12	−3.60, −0.64	89	<0.01			
Healthy	9	25	390	195	195	−3.07	−4.29, −1.85	92	<0.01	15.36	93.5	<0.01
Injury	5	10	123	47	76	0.02	−0.93, 0.97	77	0.79			
*L*_m_	13	40	455	190	265	2.14	1.42, 2.86	81	<0.01			
<PND 15	4	14	142	56	86	0.99	0.30, 1.67	56	<0.01	8.47	88.2	<0.01
≥PND 15	10	26	313	134	179	2.92	1.81, 4.03	85	<0.01			
Healthy	11	31	336	145	191	3.00	2.23, 3.76	71	<0.01	18.04	94.5	<0.01
Injury	4	9	119	45	74	−0.16	−1.40, 1.08	86	0.8			
Wall thickness	6	25	203	98	105	−0.42	−0.86, 0.02	47	0.06			
<PND 15	5	12	103	51	52	−1.04	−1.89, 0.19	66	0.02	4.62	78.3	0.03
≥PND 15	4	13	100	47	53	−0.01	−0.42, 0.41	0	0.98			
Airspace volume	7	25	206	102	104	0.40	−0.06, 0.85	49	0.09			
<PND 15	6	15	118	58	60	0.49	−0.10, 1.08	43	0.10	0.20	0	0.65
≥PND 15	4	10	88	44	44	0.27	−0.48, 1.02	59	0.48			
Surface area	13	45	433	203	230	−1.51	−2.09, −0.93	75	<0.01			
<PND 15	6	17	156	64	92	−0.88	−1.86, 0.09	72	0.08	2.27	55.9	0.13
≥PND 15	11	28	277	139	138	−1.81	−2.51, −1.10	75	<0.01			
Healthy	13	41	396	183	213	−1.55	−2.16, −0.95	74	<0.01	0.10	0	0.75
Injury	3	4	37	20	17	−1.17	−3.46, 1.12	84	0.32			

Subgroups: age at evaluation of outcome measure <15 PND or ≥15 PND and healthy or animals with lung injury.

*N* total number, *SMD* standardized mean difference, *CI* confidence interval, *I*^2^ heterogeneity, *PND* postnatal day, *RAC* radial alveolar count, *L*_*m*_ alveolar mean chord length.

#### Body weight (growth)

Meta-analysis showed that newborn animals exposed to corticosteroids had a decrease in body weight compared to controls (SMD −1.72 [95% CI −2.08, −1.35], *p* < 0.01, *I*^2^ = 85%, 22 studies; 88 comparisons; 1487 animals). Subgroup analysis for age at evaluation of outcome showed a larger decrease in body weight in newborn animals analyzed before 15 days of age (Supplement 5, subgroup difference *p* < 0.00001, *I*^2^ = 97.1%). There was no subgroup difference in the effect of corticosteroids between healthy newborn animals and newborn animals with lung injury (Table 2).

#### Lung volume and -weight

There was no effect of corticosteroids on lung volume and lung weight, nor was there an effect of corticosteroids on lung volume based on age at evaluation of outcome (Table 2). Due to a lack of studies no subgroup analysis was possible for the subgroup age for lung weight, nor was it possible for the subgroup healthy versus injured newborn animals for both lung weight and -volume.

#### Radial alveolar count (RAC)

**Fig. 2:**
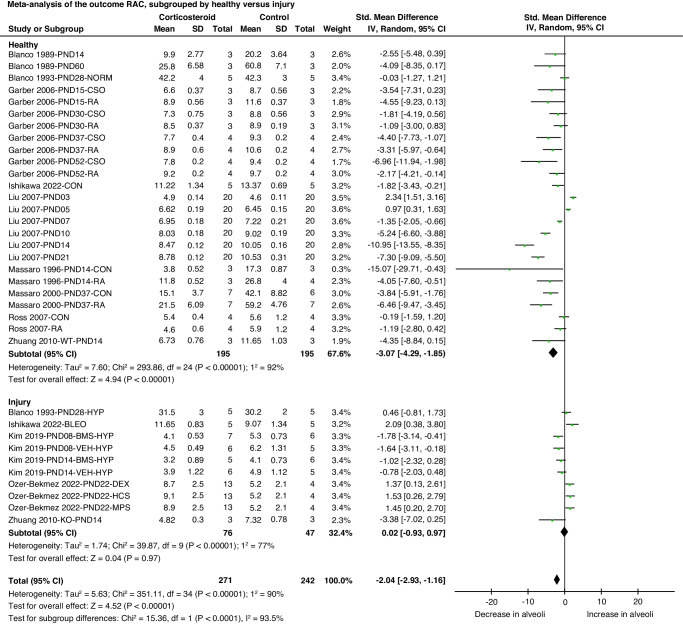
Forest plot of meta-analysis comparing the effect of corticosteroids on the number of alveoli (RAC) for the subgroup lung injury. Effect size is calculated as Standardized Mean Difference (SMD), with a 95% confidence interval (95% CI) in a random effects model. RAC radial alveolar count, PND postnatal day, NORM normoxia, CSO cottonseed oil, RA retinoic acid, CON control, WT wild-type, HYP hyperoxia, BLEO bleomycin, BMS betamethasone, VEH vehicle, DEX dexamethasone, HCS hydrocortisone, MPS methylprednisolone, KO knock out.

RAC is a measure of the number of alveoli. Meta-analysis showed that corticosteroid treatment resulted in a decrease in RAC compared to the control group (SMD −2.04 [95% CI −2.93, −1.16], *P* < 0.01, *I*^2^ = 90%; 11 studies; 35 comparisons; 513 animals). Subgroup analysis based on age at evaluation of outcome showed no differences. However, subgroup analysis based on health status showed a larger reduction in RAC in healthy newborn animals compared to newborn animals with lung injury (Fig. 2, test for subgroup difference *P* < 0.0001, *I*^2^ = 93.5%).

#### Alveolar mean chord length (L_m_)

**Fig. 3:**
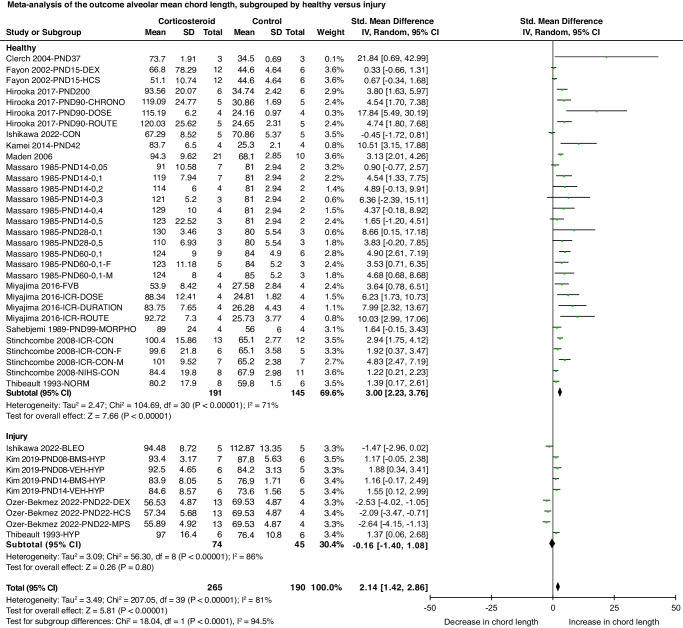
Forest plot of meta-analysis comparing the effect of corticosteroids on the alveolar mean chord length (*L*_m_) for the subgroup lung injury. Effect size is calculated as Standardized Mean Difference (SMD), with a 95% confidence interval (95% CI) in a random effects model. PND postnatal day, DEX dexamethasone, HCS hydrocortisone, CON control, F female, M male, NORM normoxia, BLEO bleomycin, BMS betamethasone, HYP hyperoxia, VEH vehicle, MPS methylprednisolone. CHRONO, DOSE, ROUTE, DURATION, and MORPHO: different experimental protocols. FVB, ICR, and NIH: different mouse strains.

*L*_m_ is a measure of the acinar air space complex (alveoli and alveolar ducts combined). Meta-analysis showed that corticosteroid exposure increased *L*_m_ (SMD 2.14 [95% CI 1.42, 2.86], *P* < 0.01, *I*^2^ = 81%; 13 studies; 40 comparisons; 455 animals). A subgroup difference was found based on age at evaluation of outcome (test for subgroup difference, *P* < 0.01, *I*^2^ = 88.2%) showing that analysis after 15 days of age revealed a more profound increase in *L*_m_ than analysis before 15 days. Furthermore, subgroup analysis comparing the effect of corticosteroids in healthy versus injured newborn animals showed that corticosteroids had no effect on *L*_m_ in the injured group (Fig. 3, test for subgroup difference, *P* < 0.01, *I*^2^ = 94.5%).

#### Wall thickness

Wall thickness is a measure of the width of inter-alveolar septal walls. Overall, meta-analysis showed no difference in wall thickness between newborn animals exposed to corticosteroids or the control group. A subgroup difference was however found based on age at evaluation of outcome showing a larger decrease in wall thickness in newborn animals analyzed before 15 days of age compared to analysis after 15 days (test for subgroup difference, *P* = 0.03, *I*^2^ = 78.3%). Subgroup comparison based on health status was not possible due to a paucity of study data (Table 2).

#### Airspace volume and lung surface area

No apparent effect of corticosteroid treatment was found for the outcome airspace volume. Subgroup analysis based on age at evaluation of outcome did not change this finding while subgroup analysis based on health status was not possible due to a lack of study data. For lung surface area meta-analysis showed that surface area was decreased in newborn animals exposed to corticosteroids compared to controls (SMD −1.51 [95% CI −2.09, −0.93], *p* < 0.01, *I*^2^ 75%, 13 studies; 45 comparisons; 433 animals). No subgroup differences based on age at evaluation of outcome or health status were found for lung surface area (Table 2).

## Discussion

This systematic review provides insight into the size, variability, and validity of the preclinical evidence of the effects of postnatal systemic corticosteroids on lung development in newborn animals in order to support its use in preterm infants at risk of BPD. Overall study quality was mediocre and the risk of bias was unclear in all domains because of poor reporting. Meta-analyses showed that corticosteroids in healthy conditions had a negative impact on lung development as well as on body weight of newborn animals. Administration of corticosteroids to healthy animals resulted in alveolar simplification shown as a persistent decrease in the number of alveoli (RAC) and surface area, a persistent increase in *L*_m_, and an early transient decrease in alveolar septal wall thickness. Conversely, in animals with lung injury corticosteroids appeared to have little effect on most outcomes; RAC and surface area did not decrease, nor did *L*_m_ increase compared to newborn animals with lung injury but without corticosteroid treatment. A few findings are noteworthy:

First, alveolar simplification as a result of postnatal systemic corticosteroids in healthy newborn animals is concerning. Hypothetically, this finding could be explained by a (temporary) increase in lung maturation with an increase in cellular differentiation and thinning of alveolar septal walls at the expense of proliferation (septation), eventually resulting in a structurally simplified and smaller lung.^75–77^ Acceleration of maturation in its broadest sense, including upregulation of surfactant production and its release and improvement of hemodynamics is essentially also the key rationale for the use of *prenatal* corticosteroids in imminent preterm labour.^78^

Second, the reduced effect of corticosteroids in newborn animals with lung injury is puzzling. One explanation for the absence of differences in RAC and *L*_m_ could be the fact that lung injury by itself, especially hyperoxia, causes alveolar simplification and corticosteroids simply do not aggravate (or ameliorate) this effect.^26^ Another explanation could be that the inflicted injury is so overwhelming that any effect of corticosteroids is completely overshadowed. Interestingly, these different effects of corticosteroids in healthy and injured lungs may in part explain the results from human randomized controlled trials, which showed that the effect of corticosteroids on the reduction of BPD is less profound with prophylactic treatment.^14^ A prophylactic treatment strategy unavoidably includes preterm infants with less severe lung injury (i.e., healthier lungs) who are at lower risk of a protracted course of invasive ventilation. Hence, the reduction in days on invasive ventilation will be modest and we speculate that instead the direct (negative) effects of corticosteroids on lung development will dominate and might even contribute to some form of alveolar simplification in these infants.

Third, subgroup analysis on age at evaluation differentiated between acute and long-term (persistence of) effects of postnatal corticosteroids. This analysis showed that the decrease in body weight and thinning of the alveolar septal walls was most notable during peak alveolarization (<15 days of age) and resolved (septal wall thinning) or partially came back up (body weight) thereafter (≥15 days of age). On the contrary, the effect on *L*_m_ (*L*_m_ increased) was visible before 15 days of age and *increased* with time (≥15 days of age). The effect on surface area (decreased) was only manifested after 15 days of age. The decrease in RAC was an early (<15 days of age) effect that did not resolve with time. Together these observations suggest that postnatal systemic corticosteroids in newborn animals have an early negative impact on body weight and lung structure that results in persistent alveolar simplification. The effect on thinning of the alveolar walls was not persistent and disappeared when analyzed after 15 days. This finding seems to be in line with the clinical observation that courses of pre- and postnatal corticosteroids improve the preterm infant’s lung condition as revealed by a reduced need for oxygen and respiratory support but that this effect wears off over time.^78,79^

Fourth, human randomized controlled trials have shown clear evidence for a reduction in BPD with (timed and targeted) postnatal corticosteroid treatment in preterm infants. The underlying mechanism for this effect is believed to be a reduction of inflammation which improves lung condition and allows for earlier weaning of invasive ventilation and supplemental oxygen, thereby preventing ongoing inflammation with further lung injury and subsequent development of BPD.^9^ We speculate that a maturational effect of corticosteroids could also be part of this underlying mechanism especially the temporary thinning of alveolar walls. Unfortunately, we found no evidence for either of these explanatory mechanisms in this systematic review due to a paucity of studies reporting on (comparable) inflammatory markers or on alveolar wall thickness in newborn animals with lung injury and corticosteroid treatment.

Overall, in spite of the widespread use of postnatal systemic corticosteroids and the great clinical controversy of treating versus withholding corticosteroids for preterm infants, we found surprisingly limited preclinical research on this topic, especially the use of corticosteroids in lung injury models. The limited number of available studies prevented us from performing a publication bias analysis, and it is therefore uncertain to which extent this bias may be influencing our meta-analyses results. Furthermore, it is important to realize that subgroup analyses are hypothesis-generating, and further studies are needed to confirm (or reject) our findings. The identified small amount of evidence is tremendously heterogeneous in terms of used treatment regimens, which limits our ability to assess the impact of clinically relevant modifiers such as dose, duration, and timing of corticosteroid efficacy through subgroup analysis or meta-regression. Also, nearly all studies (98%) used dexamethasone, while a recently published French cohort study showed that the only corticosteroid given to preterm infants from 2017 to 2021 was hydrocortisone.^1^ Comparing outcomes across studies was equally challenging because different methods of measurement to assess RAC, *L*_m_, wall thickness, lung surface area, and airspace volume were used among studies.

Additionally, we expected to find more common outcomes related to inflammation, lung matrix, and lung function as inflammation, with an imbalance in pro-inflammatory and anti-inflammatory cytokines is assumed to play a crucial role in the development of BPD, and compromised lung function and impaired exercise tolerance are significant long-term sequelae in preterm infants surviving with BPD.^80–85^

The used animal models had several limitations which compromised their external validity for BPD as well. For example, models combining more than two injurious insults are lacking, while (evolving) BPD is of multifactorial origin.^5,8^ Also, most studies exposed newborn animals to hyperoxia as high as 50–97% oxygen and some used hyperoxia exposure times as long as 3 weeks, which significantly exceeds the oxygen exposure of human preterm infants in contemporary clinical settings. Furthermore, we found only one study that exposed animals to LPS before administering corticosteroids, while sepsis is a major risk factor for BPD.^86^

Some studies used a 10-day course of dexamethasone which resembles the human (modified) Dexamethasone: A Randomized Trial (DART) protocol that is used in many clinical units.^87,88^ However, direct comparisons of these studies with the human situation is complex since lung development in rodents progresses much faster than in humans. A 10-day course in newborn rats or mice comprises most saccular and half of the total alveolar stage of lung development, while a 10-day course in an extremely preterm infant of 26 weeks comprises only the beginning of the saccular stage of lung development.^89,90^ Also, more differences exist between rodent and human lung development. For example, while term rodents and extremely preterm infants are both born in the saccular stage of lung development, term rodents are surfactant sufficient while extreme preterm infants are not.^89,90^ We therefore emphasize that direct comparisons of corticosteroid treatment regimes in experimental rodent studies on lung development to the human situation should be interpreted with caution.

Furthermore, new BPD with alveolar simplification is of highest risk in the most extreme preterm infants which is not well translated in a term rodent model. Iatrogenic prematurity is however possible in large (non-rodent) animals and several research groups have studied the effects of prenatal corticosteroids on respiratory distress syndrome in preterm animal models.^91–93^ Unfortunately, studies that administered postnatal corticosteroids to large (preterm) animals did not meet the inclusion criteria of this systematic review because follow-up times were less than 24 h or corticosteroids were administered intra-tracheal instead of systemic.^94–96^

All these issues raise concerns about the translatability of the worrying experimental findings of this systematic review. Future preclinical studies should therefore minimize risks of bias, enhance reporting and methodological quality, and strive for more standardization in (measurement of) outcome parameters, corticosteroid treatment regimens, and lung injury models to enhance comparability. Also, (large) animal models should represent the current clinical setting more closely and address the multifactorial nature of BPD in order to elucidate the delicate balance between the detrimental and beneficial effects of corticosteroids on lung development and BPD.

## Conclusion

This is the first systematic review and meta-analysis of the effects of postnatal systemic corticosteroids on lung development in animal studies. We found that postnatal corticosteroids have a negative effect on body weight and lung development resulting in persistent alveolar simplification. This detrimental effect on lung structure was mainly observed in healthy animals, which might suggest that corticosteroids should only be considered in preterm infants with lung injury who are at high risk of developing BPD. We do want to emphasize that studies were extremely heterogeneous in design (for example for dosages and duration of corticosteroid treatment), had unclear quality due to insufficient methodological reporting, and used animal models not accurately representing the clinical conditions of high-risk BPD infants.

There is a need for new preclinical studies that mimic the current clinical situation more truly in multi-hit animal models. Those models should investigate different regimens and types of postnatal corticosteroids and should ideally not only focus on short-term outcomes like lung morphology and -inflammation but on long-term physiologic outcomes like lung function as well.

## Supplementary information

Supplementary material

## Data Availability

The datasets generated during and/or analyzed during the current study are available from the corresponding author upon reasonable request.

## References

[1] Iacobelli, S. et al. Postnatal corticosteroid exposure in very preterm infants: a French cohort study. *Front. Pharm.* **14**, 1170842 (2023). 10.3389/fphar.2023.1170842 PMC10113548 37089932

[2] Thebaud, B. et al. Bronchopulmonary dysplasia. *Nat. Rev. Dis. Prim.* **5**, 78 (2019). 31727986 10.1038/s41572-019-0127-7 PMC6986462

[3] Katz, T. A. et al. Severity of bronchopulmonary dysplasia and neurodevelopmental outcome at 2 and 5 years corrected age. *J. Pediatr.* **243**, 40–46.e42 (2022). 34929243 10.1016/j.jpeds.2021.12.018

[4] Morrow, L. A. et al. Antenatal determinants of bronchopulmonary dysplasia and late respiratory disease in preterm infants. *Am. J. Respir. Crit. Care Med.* **196**, 364–374 (2017). 28249118 10.1164/rccm.201612-2414OC PMC5549867

[5] Principi, N., Di Pietro, G. M. & Esposito, S. Bronchopulmonary dysplasia: clinical aspects and preventive and therapeutic strategies. *J. Transl. Med.* **16**, 36 (2018). 29463286 10.1186/s12967-018-1417-7 PMC5819643

[6] Schmidt, A. R. & Ramamoorthy, C. Bronchopulmonary dysplasia. *Paediatr. Anaesth.* **32**, 174–180 (2022). 34877749 10.1111/pan.14365

[7] Alvira, C. M. Aberrant pulmonary vascular growth and remodeling in bronchopulmonary dysplasia. *Front. Med.* **3**, 21 (2016). 10.3389/fmed.2016.00021 PMC4873491 27243014

[8] Jensen, E. A. & Schmidt, B. Epidemiology of bronchopulmonary dysplasia. *Birth Defects Res. A Clin. Mol. Teratol.* **100**, 145–157 (2014). 24639412 10.1002/bdra.23235 PMC8604158

[9] Doyle, L. W., Cheong, J. L., Hay, S., Manley, B. J. & Halliday, H. L. Late (>/= 7 Days) systemic postnatal corticosteroids for prevention of bronchopulmonary dysplasia in preterm infants. *Cochrane Database Syst. Rev.* **11**, CD001145 (2021). 34758507 10.1002/14651858.CD001145.pub5 PMC8580679

[10] Onland, W., van de Loo, M., Offringa, M. & van Kaam, A. Systemic corticosteroid regimens for prevention of bronchopulmonary dysplasia in preterm infants. *Cochrane Database Syst. Rev.* **3**, CD010941 (2023). 36912887 10.1002/14651858.CD010941.pub3 PMC10015219

[11] Jenkinson, A. C., Kaltsogianni, O., Dassios, T. & Greenough, A. Postnatal corticosteroid usage in United Kingdom and Ireland neonatal units. *Acta Paediatr.* **112**, 2503–2506 (2023). 10.1111/apa.16968 37675620

[12] Parikh, S. et al. Trends, characteristic, and outcomes of preterm infants who received postnatal corticosteroid: a cohort study from 7 high-income countries. *Neonatology* **120**, 517–526 (2023). 37166345 10.1159/000530128 PMC10614478

[13] Jensen, E. A. et al. Assessment of corticosteroid therapy and death or disability according to pretreatment risk of death or bronchopulmonary dysplasia in extremely preterm infants. *JAMA Netw. Open* **6**, e2312277 (2023). 37155165 10.1001/jamanetworkopen.2023.12277 PMC10167571

[14] Doyle, L. W., Cheong, J. L., Hay, S., Manley, B. J. & Halliday, H. L. Early (≪ 7 Days) systemic postnatal corticosteroids for prevention of bronchopulmonary dysplasia in preterm infants. *Cochrane Database Syst. Rev.* **10**, CD001146 (2021). 29063585 10.1002/14651858.CD001146.pub5 PMC6485683

[15] Onland, W., De Jaegere, A. P., Offringa, M. & van Kaam, A. Systemic corticosteroid regimens for prevention of bronchopulmonary dysplasia in preterm infants. *Cochrane Database Syst. Rev.* **1**, CD010941 (2017). 28141913 10.1002/14651858.CD010941.pub2 PMC6464844

[16] Onland, W., Offringa, M. & van Kaam, A. Late (>/= 7 Days) inhalation corticosteroids to reduce bronchopulmonary dysplasia in preterm infants. *Cochrane Database Syst. Rev.* **8**, CD002311 (2017). 28836266 10.1002/14651858.CD002311.pub4 PMC6483527

[17] Puia-Dumitrescu, M. et al. Dexamethasone, prednisolone, and methylprednisolone use and 2-year neurodevelopmental outcomes in extremely preterm infants. *JAMA Netw. Open* **5**, e221947 (2022). 35275165 10.1001/jamanetworkopen.2022.1947 PMC8917427

[18] Kilkenny, C., Browne, W. J., Cuthill, I. C., Emerson, M. & Altman, D. G. Improving bioscience research reporting: the arrive guidelines for reporting animal research. *PLoS Biol.* **8**, e1000412 (2010). 20613859 10.1371/journal.pbio.1000412 PMC2893951

[19] Page, M. J. et al. The Prisma 2020 statement: an updated guideline for reporting systematic reviews. *BMJ* **372**, n71 (2021). 33782057 10.1136/bmj.n71 PMC8005924

[20] Schneider, C. A., Rasband, W. S. & Eliceiri, K. W. Nih image to imagej: 25 years of image analysis. *Nat. Methods* **9**, 671–675 (2012). 22930834 10.1038/nmeth.2089 PMC5554542

[21] Hooijmans, C. R. et al. Syrcle’s risk of bias tool for animal studies. *BMC Med. Res. Methodol.* **14**, 43 (2014). 24667063 10.1186/1471-2288-14-43 PMC4230647

[22] Tschanz, S. A. et al. Rat lungs show a biphasic formation of new alveoli during postnatal development. *J. Appl. Physiol.* **117**, 89–95 (2014). 24764134 10.1152/japplphysiol.01355.2013

[23] Mund, S. I., Stampanoni, M. & Schittny, J. C. Developmental alveolarization of the mouse lung. *Dev. Dyn.* **237**, 2108–2116 (2008). 18651668 10.1002/dvdy.21633

[24] Bartolome, J. V., Wang, S., Greer, N. L. & Schanberg, S. M. Glucocorticoid regulation of ornithine decarboxylase in the postnatal rat lung. *Life Sci.* **64**, 895–904 (1999). 10201638 10.1016/s0024-3205(99)00015-6

[25] Blanco, L. N., Massaro, G. D. & Massaro, D. Alveolar dimensions and number: developmental and hormonal regulation. *Am. J. Physiol.* **257**, L240–L247 (1989). 2801952 10.1152/ajplung.1989.257.4.L240

[26] Blanco, L. N. & Frank, L. The formation of alveoli in rat lung during the third and fourth postnatal weeks: effect of hyperoxia, dexamethasone, and deferoxamine. *Pediatr. Res.* **34**, 334–340 (1993). 8134176 10.1203/00006450-199309000-00019

[27] Corroyer, S., Schittny, J. C., Djonov, V., Burri, P. H. & Clement, A. Impairment of rat postnatal lung alveolar development by glucocorticoids: involvement of the P21cip1 and P27kip1 cyclin-dependent kinase inhibitors. *Pediatr. Res.* **51**, 169–176 (2002). 11809910 10.1203/00006450-200202000-00008

[28] Dallas, D. V., Keeney, S. E., Mathews, M. J. & Schmalstieg, F. C. Effects of postnatal dexamethasone on oxygen toxicity in neonatal rats. *Biol. Neonate* **86**, 145–154 (2004). 15205572 10.1159/000079114

[29] Fayon, M., Jouvencel, P., Carles, D., Choukroun, M. L. & Marthan, R. Differential effect of dexamethasone and hydrocortisone on alveolar growth in rat pups. *Pediatr. Pulmonol.* **33**, 443–448 (2002). 12001277 10.1002/ppul.10108

[30] Floros, J., Phelps, D. S., Harding, H. P., Church, S. & Ware, J. Postnatal stimulation of rat surfactant protein a synthesis by dexamethasone. *Am. J. Physiol.* **257**, L137–L143 (1989). 2475036 10.1152/ajplung.1989.257.2.L137

[31] Garber, S. J. et al. Hormonal regulation of alveolarization: structure-function correlation. *Respir. Res.* **7**, 47 (2006). 16566837 10.1186/1465-9921-7-47 PMC1448204

[32] Gesche, J. et al. Rhkgf stimulates lung surfactant production in neonatal rats in vivo. *Pediatr. Pulmonol.* **46**, 882–895 (2011). 21462359 10.1002/ppul.21443

[33] Hu, J., Yu, M., Tang, Y. & Tian, Z. F. Glucocorticoid attenuates hyperoxia-induced lung injury in neonatal rat and inhibits rage and Nf-Kb expression. *Int. J. Clin. Exp. Med.* **11**, 2519–2523 (2018).

[34] Ishikawa, S. et al. A glucocorticoid-receptor agonist ameliorates bleomycin-induced alveolar simplification in newborn rats. *Pediatr. Res.* **93**, 1551–1558 (2023). 10.1038/s41390-022-02257-8 36068343

[35] Kim, Y. E., Park, W. S., Sung, D. K., Ahn, S. Y. & Chang, Y. S. Antenatal betamethasone enhanced the detrimental effects of postnatal dexamethasone on hyperoxic lung and brain injuries in newborn rats. *PLoS One* **14**, e0221847 (2019). 31469886 10.1371/journal.pone.0221847 PMC6716665

[36] le Cras, T. D. et al. Neonatal dexamethasone treatment increases the risk for pulmonary hypertension in adult rats. *Am. J. Physiol. Lung Cell Mol. Physiol.* **278**, L822–L829 (2000). 10749760 10.1152/ajplung.2000.278.4.L822

[37] Lee, H. J. et al. Effects of postnatal dexamethasone or hydrocortisone in a rat model of antenatal lipopolysaccharide and neonatal hyperoxia exposure. *J. Korean Med. Sci.* **27**, 395–401 (2012). 22468103 10.3346/jkms.2012.27.4.395 PMC3314852

[38] Lindsay, L. et al. Modulation of hyperoxia-induced Tnf-alpha expression in the newborn rat lung by thalidomide and dexamethasone. *Inflammation* **24**, 347–356 (2000). 10850856 10.1023/a:1007096931078

[39] Liu, H. C. et al. Insulin-like growth factors in lung development of neonatal rats and effect of dexamethasone and retinoic acid on their expression. *World J. Pediatr.* **3**, 55–60 (2007).

[40] Luyet, C., Burri, P. H. & Schittny, J. C. Suppression of cell proliferation and programmed cell death by dexamethasone during postnatal lung development. *Am. J. Physiol. Lung Cell Mol. Physiol.* **282**, L477–L483 (2002). 11839541 10.1152/ajplung.00406.2000

[41] Massaro, D., Teich, N., Maxwell, S., Massaro, G. D. & Whitney, P. Postnatal development of alveoli. regulation and evidence for a critical period in rats. *J. Clin. Investig.* **76**, 1297–1305 (1985). 4056033 10.1172/JCI112103 PMC424058

[42] Massaro, G. D. & Massaro, D. Development of bronchiolar epithelium in rats. *Am. J. Physiol.* **250**, R783–R788 (1986). 3706566 10.1152/ajpregu.1986.250.5.R783

[43] Massaro, D. & Massaro, G. D. Dexamethasone accelerates postnatal alveolar wall thinning and alters wall composition. *Am. J. Physiol.* **251**, R218–R224 (1986). 3740302 10.1152/ajpregu.1986.251.2.R218

[44] Massaro, G. D. & Massaro, D. Postnatal treatment with retinoic acid increases the number of pulmonary alveoli in rats. *Am. J. Physiol.* **270**, L305–L310 (1996). 8780001 10.1152/ajplung.1996.270.2.L305

[45] Massaro, G. D. & Massaro, D. Retinoic acid treatment partially rescues failed septation in rats and in mice. *Am. J. Physiol. Lung Cell Mol. Physiol.* **278**, L955–L960 (2000). 10781425 10.1152/ajplung.2000.278.5.L955

[46] Ogasawara, Y. et al. Pre- and postnatal stimulation of pulmonary surfactant protein D by in vivo dexamethasone treatment of rats. *Life Sci.* **50**, 1761–1767 (1992). 1598064 10.1016/0024-3205(92)90059-x

[47] Ozer Bekmez, B. et al. Glucocorticoids in a neonatal hyperoxic lung injury model: pulmonary and neurotoxic effects. *Pediatr. Res.* **92**, 436–444 (2022). 34725500 10.1038/s41390-021-01777-z

[48] Ross, A. C. & Ambalavanan, N. Retinoic acid combined with vitamin A synergizes to increase retinyl ester storage in the lungs of newborn and dexamethasone-treated neonatal rats. *Neonatology* **92**, 26–32 (2007). 17596734 10.1159/000100083 PMC3843127

[49] Roth-Kleiner, M., Berger, T. M., Tarek, M. R., Burri, P. H. & Schittny, J. C. Neonatal dexamethasone induces premature microvascular maturation of the alveolar capillary network. *Dev. Dyn.* **233**, 1261–1271 (2005). 15937935 10.1002/dvdy.20447

[50] Roth-Kleiner, M. et al. Neonatal steroids induce a down-regulation of tenascin-C and elastin and cause a deceleration of the first phase and an acceleration of the second phase of lung alveolarization. *Histochem. Cell Biol.* **141**, 75–84 (2014). 23912843 10.1007/s00418-013-1132-7

[51] Sahebjami, H. & Domino, M. Effects of postnatal dexamethasone treatment on development of alveoli in adult rats. *Exp. Lung Res.* **15**, 961–973 (1989). 2612450 10.3109/01902148909069638

[52] Schwyter, M., Burri, P. H. & Tschanz, S. A. Geometric properties of the lung parenchyma after postnatal glucocorticoid treatment in rats. *Biol. Neonate* **83**, 57–64 (2003). 12566685 10.1159/000067010

[53] Shimizu, H., Miyamura, K. & Kuroki, Y. Appearance of surfactant proteins, Sp-A and Sp-B, in developing rat lung and the effects of in vivo dexamethasone treatment. *Biochim. Biophys. Acta* **1081**, 53–60 (1991). 1991156 10.1016/0005-2760(91)90249-h

[54] Srinivasan, G., Bruce, E. N., Houtz, P. K. & Bruce, M. C. Dexamethasone-induced changes in lung function are not prevented by concomitant treatment with retinoic acid. *Am. J. Physiol. Lung Cell Mol. Physiol.* **283**, L275–L287 (2002). 12114188 10.1152/ajplung.00423.2001

[55] Theogaraj, E., John, C. D., Dewar, A., Buckingham, J. C. & Smith, S. F. The long-term effects of perinatal glucocorticoid exposure on the host defence system of the respiratory tract. *J. Pathol.* **210**, 85–93 (2006). 16924656 10.1002/path.2017

[56] Thibeault, D. W., Heimes, B., Rezaiekhaligh, M. & Mabry, S. Chronic modifications of lung and heart development in glucocorticoid-treated newborn rats exposed to hyperoxia or room air. *Pediatr. Pulmonol.* **16**, 81–88 (1993). 8367221 10.1002/ppul.1950160202

[57] Tsai, M. H. et al. Cd200 in growing rat lungs: developmental expression and control by dexamethasone. *Cell Tissue Res.* **359**, 729–742 (2015). 25519046 10.1007/s00441-014-2065-8

[58] Tschanz, S. A., Damke, B. M. & Burri, P. H. Influence of postnatally administered glucocorticoids on rat lung growth. *Biol. Neonate* **68**, 229–245 (1995). 8580214 10.1159/000244241

[59] Tschanz, S. A., Makanya, A. N., Haenni, B. & Burri, P. H. Effects of neonatal high-dose short-term glucocorticoid treatment on the lung: a morphologic and morphometric study in the rat. *Pediatr. Res.* **53**, 72–80 (2003). 12508084 10.1203/00006450-200301000-00014

[60] Valencia, A. M. et al. Early postnatal dexamethasone influences matrix metalloproteinase-2 and -9, and their tissue inhibitors in the developing rat lung. *Pediatr. Pulmonol.* **35**, 456–462 (2003). 12746943 10.1002/ppul.10293

[61] Veness-Meehan, K. A., Bottone, F. G. Jr. & Stiles, A. D. Effects of retinoic acid on airspace development and lung collagen in hyperoxia-exposed newborn rats. *Pediatr. Res.* **48**, 434–444 (2000). 11004232 10.1203/00006450-200010000-00004

[62] Zhang, H. et al. The angiogenic factor midkine is regulated by dexamethasone and retinoic acid during alveolarization and in alveolar epithelial cells. *Respir. Res.* **10**, 77 (2009). 19698107 10.1186/1465-9921-10-77 PMC2739515

[63] Bhatt, A. J., Amin, S. B., Chess, P. R., Watkins, R. H. & Maniscalco, W. M. Expression of vascular endothelial growth factor and Flk-1 in developing and glucocorticoid-treated mouse lung. *Pediatr. Res.* **47**, 606–613 (2000). 10813585 10.1203/00006450-200005000-00009

[64] Clerch, L. B., Baras, A. S., Massaro, G. D., Hoffman, E. P. & Massaro, D. DNA microarray analysis of neonatal mouse lung connects regulation of Kdr with dexamethasone-induced inhibition of alveolar formation. *Am. J. Physiol. Lung Cell Mol. Physiol.* **286**, L411–L419 (2004). 14607780 10.1152/ajplung.00306.2003

[65] Hirooka, S., Ueno, M., Fukuda, S., Miyajima, A. & Hirota, T. Effects of simvastatin on alveolar regeneration and its relationship to exposure in mice with dexamethasone-induced emphysema. *Biol. Pharm. Bull.* **40**, 155–160 (2017). 28154254 10.1248/bpb.b16-00637

[66] Kamei, M., Miyajima, A., Fujisawa, M., Matsuoka, Y. & Hirota, T. Effects of postnatal dexamethasone treatment on mRNA expression profiles of genes related to alveolar development in an emphysema model in mice. *J. Toxicol. Sci.* **39**, 665–670 (2014). 25056791 10.2131/jts.39.665

[67] Maden, M. Retinoids have differing efficacies on alveolar regeneration in a dexamethasone-treated mouse. *Am. J. Respir. Cell Mol. Biol.* **35**, 260–267 (2006). 16574940 10.1165/rcmb.2006-0029OC

[68] McGowan, S. E. & McCoy, D. M. Glucocorticoids retain bipotent fibroblast progenitors during alveolar septation in mice. *Am. J. Respir. Cell Mol. Biol.* **57**, 111–120 (2017). 28530121 10.1165/rcmb.2016-0376OC PMC5946667

[69] Mi, L. et al. Tissue-resident type 2 innate lymphoid cells arrest alveolarization in bronchopulmonary dysplasia. *J. Immunol. Res.* **2020**, 8050186 (2020). 33178840 10.1155/2020/8050186 PMC7648679

[70] Miyajima, A. et al. Effects of all trans-retinoic acid on alveolar regeneration in dexamethasone-induced emphysema models and its relationship to exposure in Icr and Fvb mice. *Biol. Pharm. Bull.* **39**, 927–934 (2016). 27251495 10.1248/bpb.b15-00704

[71] Ohtsu, N. et al. The effect of dexamethasone on chronic pulmonary oxygen toxicity in infant mice. *Pediatr. Res.* **25**, 353–359 (1989). 2726308 10.1203/00006450-198904000-00008

[72] Stinchcombe, S. V. & Maden, M. Retinoic acid induced alveolar regeneration: critical differences in strain sensitivity. *Am. J. Respir. Cell Mol. Biol.* **38**, 185–191 (2008). 17717321 10.1165/rcmb.2007-0252OC

[73] Zhuang, T., Zhang, M., Zhang, H., Dennery, P. A. & Lin, Q. S. Disrupted postnatal lung development in heme oxygenase-1 deficient mice. *Respir. Res.* **11**, 142 (2010). 20932343 10.1186/1465-9921-11-142 PMC2964616

[74] Town, G. I. et al. Dexamethasone treatment fails to reduce oxygen-induced lung injury in the preterm Guinea pig. effects on pulmonary inflammation and antioxidant status. *Biochem. Pharm.* **46**, 1565–1572 (1993). 8240412 10.1016/0006-2952(93)90324-p

[75] Bird, A. D., Choo, Y. L., Hooper, S. B., McDougall, A. R. & Cole, T. J. Mesenchymal glucocorticoid receptor regulates the development of multiple cell layers of the mouse lung. *Am. J. Respir. Cell Mol. Biol.* **50**, 419–428 (2014). 24053134 10.1165/rcmb.2013-0169OC

[76] Bridges, J. P. et al. Glucocorticoid regulates mesenchymal cell differentiation required for perinatal lung morphogenesis and function. *Am. J. Physiol. Lung Cell Mol. Physiol.* **319**, L239–L255 (2020). 32460513 10.1152/ajplung.00459.2019 PMC7473939

[77] Muglia, L. J. et al. Proliferation and differentiation defects during lung development in corticotropin-releasing hormone-deficient mice. *Am. J. Respir. Cell Mol. Biol.* **20**, 181–188 (1999). 9922208 10.1165/ajrcmb.20.2.3381

[78] McGoldrick, E., Stewart, F., Parker, R. & Dalziel, S. R. Antenatal corticosteroids for accelerating fetal lung maturation for women at risk of preterm birth. *Cochrane Database Syst. Rev.* **12**, CD004454 (2020). 33368142 10.1002/14651858.CD004454.pub4 PMC8094626

[79] Halbmeijer, N. M. et al. Short-term pulmonary and systemic effects of hydrocortisone initiated 7-14 days after birth in ventilated very preterm infants: a secondary analysis of a randomised controlled trial. *Arch. Dis. Child. Fetal Neonatal Ed.* **108**, 20–25 (2023). 35534184 10.1136/archdischild-2022-323882

[80] Speer, C. P. Inflammation and bronchopulmonary dysplasia: a continuing story. *Semin. Fetal Neonatal Med.* **11**, 354–362 (2006). 16702036 10.1016/j.siny.2006.03.004

[81] Balany, J. & Bhandari, V. Understanding the impact of infection, inflammation, and their persistence in the pathogenesis of bronchopulmonary dysplasia. *Front. Med.* **2**, 90 (2015). 10.3389/fmed.2015.00090 PMC4685088 26734611

[82] Papagianis, P. C., Pillow, J. J. & Moss, T. J. Bronchopulmonary dysplasia: pathophysiology and potential anti-inflammatory therapies. *Paediatr. Respir. Rev.* **30**, 34–41 (2019). 30201135 10.1016/j.prrv.2018.07.007

[83] Cheong, J. L. Y. & Doyle, L. W. An update on pulmonary and neurodevelopmental outcomes of bronchopulmonary dysplasia. *Semin. Perinatol.* **42**, 478–484 (2018). 30401478 10.1053/j.semperi.2018.09.013

[84] Sillers, L., Alexiou, S. & Jensen, E. A. Lifelong pulmonary sequelae of bronchopulmonary dysplasia. *Curr. Opin. Pediatr.* **32**, 252–260 (2020). 32084032 10.1097/MOP.0000000000000884

[85] Moschino, L., Bonadies, L. & Baraldi, E. Lung growth and pulmonary function after prematurity and bronchopulmonary dysplasia. *Pediatr. Pulmonol.* **56**, 3499–3508 (2021). 33729686 10.1002/ppul.25380 PMC8597033

[86] Lapcharoensap, W. et al. The relationship of nosocomial infection reduction to changes in neonatal intensive care unit rates of bronchopulmonary dysplasia. *J. Pediatr.* **180**, 105–109.e101 (2017). 27742123 10.1016/j.jpeds.2016.09.030

[87] Doyle, L. W. et al. Low-dose dexamethasone facilitates extubation among chronically ventilator-dependent infants: a multicenter, international, randomized, controlled trial. *Pediatrics* **117**, 75–83 (2006). 16396863 10.1542/peds.2004-2843

[88] Htun, Z. T. et al. Postnatal steroid management in preterm infants with evolving bronchopulmonary dysplasia. *J. Perinatol.* **41**, 1783–1796 (2021). 34012057 10.1038/s41372-021-01083-w PMC8133053

[89] Berger, J. & Bhandari, V. Animal models of bronchopulmonary dysplasia. the term mouse models. *Am. J. Physiol. Lung Cell Mol. Physiol.* **307**, L936–L947 (2014). 25305249 10.1152/ajplung.00159.2014 PMC4269689

[90] O’Reilly, M. & Thebaud, B. Animal models of bronchopulmonary dysplasia. the term rat models. *Am. J. Physiol. Lung Cell Mol. Physiol.* **307**, L948–L958 (2014). 25305248 10.1152/ajplung.00160.2014

[91] Yoder, B. A. & Coalson, J. J. Animal models of bronchopulmonary dysplasia. the preterm baboon models. *Am. J. Physiol. Lung Cell Mol. Physiol.* **307**, L970–L977 (2014). 25281639 10.1152/ajplung.00171.2014 PMC4269686

[92] D’Angio, C. T. & Ryan, R. M. Animal models of bronchopulmonary dysplasia. the preterm and term rabbit models. *Am. J. Physiol. Lung Cell Mol. Physiol.* **307**, L959–L969 (2014). 25326582 10.1152/ajplung.00228.2014

[93] Albertine, K. H. Utility of large-animal models of Bpd: chronically ventilated preterm lambs. *Am. J. Physiol. Lung Cell Mol. Physiol.* **308**, L983–L1001 (2015). 25770179 10.1152/ajplung.00178.2014 PMC4437012

[94] Hillman, N. H. et al. Antenatal and postnatal corticosteroid and resuscitation induced lung injury in preterm sheep. *Respir. Res.* **10**, 124 (2009). 20003512 10.1186/1465-9921-10-124 PMC2802354

[95] Digeronimo, R. J. et al. Mechanical ventilation down-regulates surfactant protein a and keratinocyte growth factor expression in premature rabbits. *Pediatr. Res.* **62**, 277–282 (2007). 17622950 10.1203/PDR.0b013e3181256aeb

[96] Hillman, N. H. et al. Dose of budesonide with surfactant affects lung and systemic inflammation after normal and injurious ventilation in preterm lambs. *Pediatr. Res.* **88**, 726–732 (2020). 32066138 10.1038/s41390-020-0809-6 PMC8717708

