# Supplementary material for: Effects of postnatal corticosteroids on lung development in newborn animals. A systematic review
Source: Pediatr Res. 2024 Mar 16;96(5):1141–52. doi: 10.1038/s41390-024-03114-6 (PMC11522003; doi:10.1038/s41390-024-03114-6)
Supplement: Supplementary file 1 — Supplementary material [file 41390_2024_3114_MOESM1_ESM.pdf]

Supplementary material

Supplement 1. PRISMA 2020 checklist*.

Section and Topic	Item #	Checklist item	Location where item is reported
**TITLE**			
Title	1	Identify the report as a systematic review.	Page 1
**ABSTRACT**			
Abstract	2	See the PRISMA 2020 for Abstracts checklist.	Page 3
**INTRODUCTION**			
Rationale	3	Describe the rationale for the review in the context of existing knowledge.	Page 4
Objectives	4	Provide an explicit statement of the objective(s) or question(s) the review addresses.	Page 5
**METHODS**			
Eligibility criteria	5	Specify the inclusion and exclusion criteria for the review and how studies were grouped for the syntheses.	Page 5-6
Information sources	6	Specify all databases, registers, websites, organisations, reference lists and other sources searched or consulted to identify studies. Specify the date when each source was last searched or consulted.	Page 5
Search strategy	7	Present the full search strategies for all databases, registers and websites, including any filters and limits used.	Supplement 2
Selection process	8	Specify the methods used to decide whether a study met the inclusion criteria of the review, including how many reviewers screened each record and each report retrieved, whether they worked independently, and if applicable, details of automation tools used in the process.	Page 6
Data collection process	9	Specify the methods used to collect data from reports, including how many reviewers collected data from each report, whether they worked independently, any processes for obtaining or confirming data from study investigators, and if applicable, details of automation tools used in the process.	Page 6
Data items	10a	List and define all outcomes for which data were sought. Specify whether all results that were compatible with each outcome domain in each study were sought (e.g. for all measures, time points, analyses), and if not, the methods used to decide which results to collect.	Page 7
	10b	List and define all other variables for which data were sought (e.g. participant and intervention characteristics, funding sources). Describe any assumptions made about any missing or unclear information.	Page 6
Study risk of bias assessment	11	Specify the methods used to assess risk of bias in the included studies, including details of the tool(s) used, how many reviewers assessed each study and whether they worked independently, and if applicable, details of automation tools used in the process.	Page 7
Effect measures	12	Specify for each outcome the effect measure(s) (e.g. risk ratio, mean difference) used in the synthesis or presentation of results.	Page 7-8
Synthesis methods	13a	Describe the processes used to decide which studies were eligible for each synthesis (e.g. tabulating the study intervention characteristics and comparing against the planned groups for each synthesis (item #5)).	Page 7-8
	13b	Describe any methods required to prepare the data for presentation or synthesis, such as handling of missing summary statistics, or data conversions.	-
	13c	Describe any methods used to tabulate or visually display results of individual studies and syntheses.	Page 7
	13d	Describe any methods used to synthesize results and provide a rationale for the choice(s). If meta-analysis was performed, describe the model(s), method(s) to identify the presence and extent of statistical heterogeneity, and software package(s) used.	Page 7
	13e	Describe any methods used to explore possible causes of heterogeneity among study results (e.g. subgroup analysis, meta-regression).	Page 8
	13f	Describe any sensitivity analyses conducted to assess robustness of the synthesized results.	-
Reporting bias assessment	14	Describe any methods used to assess risk of bias due to missing results in a synthesis (arising from reporting biases).	-
Certainty assessment	15	Describe any methods used to assess certainty (or confidence) in the body of evidence for an outcome.	-

Section and Topic	Item #	Checklist item	Location where item is reported
**RESULTS**			
Study selection	16a	Describe the results of the search and selection process, from the number of records identified in the search to the number of studies included in the review, ideally using a flow diagram.	Supplement 3
	16b	Cite studies that might appear to meet the inclusion criteria, but which were excluded, and explain why they were excluded.	Supplement 3
Study characteristics	17	Cite each included study and present its characteristics.	Table 1
Risk of bias in studies	18	Present assessments of risk of bias for each included study.	-
Results of individual studies	19	For all outcomes, present, for each study: (a) summary statistics for each group (where appropriate) and (b) an effect estimate and its precision (e.g. confidence/credible interval), ideally using structured tables or plots.	Table 2
Results of syntheses	20a	For each synthesis, briefly summarise the characteristics and risk of bias among contributing studies.	Page 11-12
	20b	Present results of all statistical syntheses conducted. If meta-analysis was done, present for each the summary estimate and its precision (e.g. confidence/credible interval) and measures of statistical heterogeneity. If comparing groups, describe the direction of the effect.	Table 2
	20c	Present results of all investigations of possible causes of heterogeneity among study results.	Page 11-12
	20d	Present results of all sensitivity analyses conducted to assess the robustness of the synthesized results.	-
Reporting biases	21	Present assessments of risk of bias due to missing results (arising from reporting biases) for each synthesis assessed.	Page 8
Certainty of evidence	22	Present assessments of certainty (or confidence) in the body of evidence for each outcome assessed.	-
**DISCUSSION**			
Discussion	23a	Provide a general interpretation of the results in the context of other evidence.	Page 13-18
	23b	Discuss any limitations of the evidence included in the review.	Page 13-18
	23c	Discuss any limitations of the review processes used.	-
	23d	Discuss implications of the results for practice, policy, and future research.	Page 19
**OTHER INFORMATION**			
Registration and protocol	24a	Provide registration information for the review, including register name and registration number, or state that the review was not registered.	Page 5
	24b	Indicate where the review protocol can be accessed, or state that a protocol was not prepared.	Page 5
	24c	Describe and explain any amendments to information provided at registration or in the protocol.	Page 5
Support	25	Describe sources of financial or non-financial support for the review, and the role of the funders or sponsors in the review.	Page 33
Competing interests	26	Declare any competing interests of review authors.	Page 33
Availability of data, code and other materials	27	Report which of the following are publicly available and where they can be found: template data collection forms; data extracted from included studies; data used for all analyses; analytic code; any other materials used in the review.	Page 19

From: Page MJ et al. The PRISMA 2020 statement: an updated guideline for reporting systematic reviews. BMJ 2021;372:n71. doi: 10.1136/bmj.n71.[19]

**Supplement 2.** Search terms in PubMed and EMBASE (via OVID).	
**PubMed**	
Corticosteroids	Adrenal cortex hormones[MeSH] OR Adrenal cortex hormone[tiab] OR Adrenal cortex hormones[tiab] OR adrenal cortical hormone[tiab] OR adrenal cortical hormones[tiab] OR adrenal cortical steroid[tiab] OR adrenal cortical steroids[tiab] OR adrenal steroid[tiab] OR adrenal steroids[tiab] OR adrenal steroid hormones[tiab] OR adreno cortical steroid[tiab] OR adreno cortical steroids[tiab] OR adreno corticosteroid[tiab] OR adreno corticosteroids[tiab] OR adrenocortical hormone[tiab] OR adrenocortical hormones[tiab] OR adrenocortical steroid[tiab] OR adrenocortical steroids[tiab] OR adrenocorticosteroid[tiab] OR adrenocorticosteroids[tiab] OR Alclometasone[tiab] OR Aldosterone[tiab] OR Amcinonide[tiab] OR Aristocort[tiab] OR Aristospan[tiab] OR Azmacort[tiab] OR Beclomethasone[tiab] OR Beclomethasone[MeSH] OR Beclometasone[tiab] OR Betamethasone [MeSH] OR Betamethasone[tiab] OR Betametasone[tiab] OR Budesonide[tiab] OR Budesonide [MeSH] OR Butixocort[tiab] OR Celestone[tiab] OR 'Chlormadinone acetate'[tiab] OR Chloroprednisone[tiab] OR ciclesonide[tiab] OR Clobetasol[MeSH] OR Clobetasol[tiab] OR Clobetasone[tiab] OR Clocortolone[tiab] OR Cloprednol[tiab] OR Cloticasone[tiab] OR Colocort[tiab] OR Cortan[tiab] OR Cortancyl[tiab] OR Cortef[tiab] OR Cortenema[tiab] OR Cortexolone[tiab] OR cortical steroid[tiab] OR cortical steroids[tiab] OR cortico steroid[tiab] OR cortico steroids[tiab] OR Corticoid[tiab] OR Corticoids[tiab] OR Corticosteroid[tiab] OR Corticosteroids[tiab] OR Corticosterone[tiab] OR Corticotropin[tiab] OR Cortifen[tiab] OR Cortifoam[tiab] OR Cortisol[tiab] OR Cortisone[tiab] OR Cortivazol[tiab] OR Cortobenzolone[tiab] OR Cortodoxone[tiab] OR Cortone Acetate[tiab] OR Cortril[tiab] OR Cotolone[tiab] OR Cyproterone acetate[tiab] OR Dacortin[tiab] OR Decadron[tiab] OR Decortin[tiab] OR Decortisyl[tiab] OR Deflazacort[tiab] OR Dehydrocortisone[tiab] OR Delta-Cortisone[tiab] OR Deltasone[tiab] OR Deoxycorticosterone[tiab] OR Deoxycortisol[tiab] OR Deoxycortisone[tiab] OR Deoxycortone[tiab] OR DepoMedrol[tiab] OR Depo-Medrol[tiab] OR dermocorticosteroid[tiab] OR dermocorticosteroids[tiab] OR Desonide[tiab] OR Desoximetasone[tiab] OR Desoximetasone[MeSH] OR Desoximethasone[tiab] OR Desoxycorticosterone acetate[tiab] OR Desoxycortone[tiab] OR Dexametasone[tiab] OR Dexamethasone[tiab] OR Dexamethasone[MeSH] OR Dexasone[tiab] OR Dexpak[tiab] OR Dichlorisone[tiab] OR Diflorasone[tiab] OR Difluocortolone[tiab] OR Diflucortolone [MeSH] OR Difluprednate[tiab] OR Drocinonide phosphate[tiab] OR Emflaza[tiab] OR Encorton[tiab] OR Encortone[tiab] OR Entocort[tiab] OR Epicortisol[tiab] OR Etiprednol dicloacetate[tiab] OR Fluclorolone[tiab] OR Flucloronide[tiab] OR 'Fludrocortisone acetate'[tiab] OR Fludrocortisone[tiab] OR Fludroxycortide[tiab] OR Flumetasone[tiab] OR Flumethasone[tiab] OR Flumethasone[MeSH] OR Flunisolide[tiab] OR 'fluocinolone acetonide'[tiab] OR fluocinolone[tiab] OR Fluocinonide[tiab] OR Fluocinonide[MeSH] OR

	Fluocortin[tiab] OR Fluocortolone[tiab] OR Fluocortolone[MeSH] OR Fluoroformylone[tiab] OR Fluorometolone[tiab] OR Fluorometholone[tiab] OR Fluorometholone[MeSH] OR Flupamesone[tiab] OR Fluperolone[tiab] OR Fluprednidene[tiab] OR Fluprednisolone[tiab] OR Fluprednisolone[MeSH] OR Flurandrenolide[tiab] OR Flurandrenolone[tiab] OR Flurandrenolone[MeSH] OR Fluticasone-Salmeterol Drug Combination[MeSH] OR Fluticasone[tiab] OR Formocortal[tiab] OR Glucocorticoid[tiab] OR Glucocorticoids[tiab] OR Glucocorticoids[Mesh] OR Halcinonide[tiab] OR Halobetasol[tiab] OR Halometasone[tiab] OR Halopredone acetate[tiab] OR Halopredone diacetate[tiab] OR HiDex[tiab] OR Hydrocort[tiab] OR Hydrocortamate[tiab] OR Hydrocortisone[tiab] OR hydroxycorticosteroid[tiab] OR hydroxycorticosteroids[tiab] OR Hydroxypregnenolone[tiab] OR Hydroxyprogesterone[tiab] OR Icometasone enbutate[tiab] OR Isoflupredone acetate[tiab] OR Kenalog-10[tiab] OR Kenalog-40[tiab] OR Kenalog[tiab] OR Ketoprogesterone[tiab] OR Liquid Pred[tiab] OR Loteprednol[tiab] OR Medrogestone[tiab] OR Medrol[tiab] OR Medroxyprogesterone acetate[tiab] OR Medrysone[tiab] OR Megestrol acetate[tiab] OR Megestrol acetate[MeSH] OR Melengestrol[tiab] OR Meprednisone[tiab] OR Methapred[tiab] OR Metapred[tiab] OR Methylpred[tiab] OR Methylprednisolone[tiab] OR Methylprednisolone[MeSH] OR Metylprednisolone[tiab] OR Methylprednisolone Hemisuccinate [MeSH] OR Meticorten[tiab] OR Mometasone furoate[tiab] OR Mometasone[tiab] OR Nicocortonide acetate[tiab] OR Nicocortonide[tiab] OR Orapred[tiab] OR Orasone[tiab] OR Panafcort[tiab] OR Panasol[tiab] OR Paramethasone[tiab] OR Paramethasone[MeSH] OR Parametasone[tiab] OR PediaPred[tiab] OR Prednazoline[tiab] OR Prednicarbate[tiab] OR Prednicot[tiab] OR Prednidib[tiab] OR Prednimustine[tiab] OR Prednisol[tiab] OR Prednisole[tiab] OR Prednisolene[tiab] OR Prednisollone[tiab] OR Prednisolne[tiab] OR Prednisoloe[tiab] OR Prednisolole[tiab] OR Prednisolon[tiab] OR Prednisolona[tiab] OR Prednisolonacetate[tiab] OR Prednisolonate[tiab] OR Prednisolone[tiab] OR Prednisolone[MeSH] OR Prednisolonebisguanylhydrazone[tiab] OR Prednisolonebisuccinate[tiab] OR Prednisolonecarboxylate[tiab] OR Prednisolonecarboxylates[tiab] OR Prednisolones[tiab] OR Prednisolonesodium[tiab] OR Prednisolonester[tiab] OR Prednisolonethe[tiab] OR Prednisolono[tiab] OR Prednisolonon[tiab] OR Prednisolonum[tiab] OR Prednisolum[tiab] OR Prednisolut[tiab] OR Prednisolute[tiab] OR Prednisom[tiab] OR Prednisome[tiab] OR Prednison[tiab] OR Prednisona[tiab] OR Prednisone[tiab] OR Prednisonlone[tiab] OR Prednisone[MeSH] OR Prednisonole[tiab] OR Prednisonum[tiab] OR Prednisonums[tiab] OR Prednisoone[tiab] OR Prednylidene[tiab] OR Pregnenolone[tiab] OR Prelone[tiab] OR Progesterone[tiab] OR Pronisone[tiab] OR Rayos[tiab] OR Rectodelt[tiab] OR Rimexolone[tiab] OR Rofleponide palmitate[tiab] OR Segesterone[tiab] OR Solu-Cortef[tiab] OR Sone[tiab] OR Sterapred[tiab] OR Steroids[MeSH] OR Steroids[tiab] OR Ticabesone[tiab] OR Timobesone acetate[tiab] OR Tixocortol[tiab] OR Tobradex[tiab] OR Tobramycin, Dexamethasone Drug Combination[MeSH] OR Triamcinolone[tiab] OR Triamcinolone[MeSH] OR Triamcinolone Acetonide [MeSH] OR Trilostane[tiab] OR

	Uceris[tiab] OR Ulobetasol[tiab] OR Ultracorten[tiab] OR Winpred[tiab] OR Zilretta[tiab]
Lung	lung[MeSH] OR lung[tiab] OR lungs[tiab] OR alveol[tiab] OR alveola[tiab] OR alveolae[tiab] OR alveolaer[tiab] OR alveolair[tiab] OR alveolaire[tiab] OR alveolar[tiab] OR alveolararterial[tiab] OR alveolarcapillary[tiab] OR alveolarepithelial[tiab] OR alveolarepithelium[tiab] OR alveolargenesis[tiab] OR alveolarisation[tiab] OR alveolarization[tiab] OR alveolarized[tiab] OR alveolarterial[tiab] OR alveolary[tiab] OR alveolas[tiab] OR alveole[tiab] OR alveolear[tiab] OR alveoler[tiab] OR alveoles[tiab] OR alveoli[tiab] OR alveolia[tiab] OR alveoliar[tiab] OR alveolies[tiab] OR alveolis[tiab] OR alveolization[tiab] OR alveolli[tiab] OR alveolo[tiab] OR alveoloarterial[tiab] OR alveolobronchial[tiab] OR alveolobronchiolar[tiab] OR alveolocapillary[tiab] OR alveolocapillay[tiab] OR alveolocyte[tiab] OR alveolocytes[tiab] OR alveologenesis[tiab] OR alveoloid[tiab] OR alveolointerstial[tiab] OR alveolointerstitial[tiab] OR alveolopathies[tiab] OR alveolopathy[tiab] OR alveoloseptal[tiab] OR alveolous[tiab] OR alveolovascular[tiab] OR alveolo-vascular[tiab] OR alveolus[tiab] OR alveoluslike[tiab] OR airspace[tiab] OR airspaces[tiab] OR air-space[tiab] OR air-spaces[tiab] OR Pulmonary[tiab]
Animal	"animal experimentation"[MeSH Terms] OR "models, animal"[MeSH Terms] OR "invertebrates"[MeSH Terms] OR "Animals"[Mesh:noexp] OR "animal population groups"[MeSH Terms] OR "chordata"[MeSH Terms:noexp] OR "chordata, nonvertebrate"[MeSH Terms] OR "vertebrates"[MeSH Terms:noexp] OR "amphibians"[MeSH Terms] OR "birds"[MeSH Terms] OR "fishes"[MeSH Terms] OR "reptiles"[MeSH Terms] OR "mammals"[MeSH Terms:noexp] OR "primates"[MeSH Terms:noexp] OR "artiodactyla"[MeSH Terms] OR "carnivora"[MeSH Terms] OR "cetacea"[MeSH Terms] OR "chiroptera"[MeSH Terms] OR "elephants"[MeSH Terms] OR "hyraxes"[MeSH Terms] OR "insectivora"[MeSH Terms] OR "lagomorpha"[MeSH Terms] OR "marsupialia"[MeSH Terms] OR "monotremata"[MeSH Terms] OR "perissodactyla"[MeSH Terms] OR "rodentia"[MeSH Terms] OR "scandentia"[MeSH Terms] OR "sirenia"[MeSH Terms] OR "haplorhini"[MeSH Terms:noexp] OR "strepsirhini"[MeSH Terms] OR "platyrrhini"[MeSH Terms] OR "tarsii"[MeSH Terms] OR "catarrhini"[MeSH Terms:noexp] OR "cercopithecidae"[MeSH Terms] OR "hylobatidae"[MeSH Terms] OR "hominidae"[MeSH Terms:noexp] OR "gorilla gorilla"[MeSH Terms] OR "pan paniscus"[MeSH Terms] OR "pan troglodytes"[MeSH Terms] OR "pongo pygmaeus"[MeSH Terms]<br><br>OR<br><br>(animals[tiab] OR animal[tiab] OR mice[Tiab] OR mus[Tiab] OR mouse[Tiab] OR murine[Tiab] OR woodmouse[tiab] OR rats[Tiab] OR rat[Tiab] OR murinae[Tiab] OR muridae[Tiab] OR cottonrat[tiab] OR cottonrats[tiab] OR hamster[tiab] OR hamsters[tiab] OR cricetinae[tiab] OR rodentia[Tiab] OR rodent[Tiab] OR

	rodents[Tiab] OR pigs[Tiab] OR pig[Tiab] OR swine[tiab] OR swines[tiab] OR piglets[tiab] OR piglet[tiab] OR boar[tiab] OR boars[tiab] OR “sus scrofa”[tiab] OR ferrets[tiab] OR ferret[tiab] OR polecat[tiab] OR polecats[tiab] OR “mustela putorius”[tiab] OR “guinea pigs”[Tiab] OR “guinea pig”[Tiab] OR cavia[Tiab] OR callithrix[Tiab] OR marmoset[Tiab] OR marmosets[Tiab] OR cebuella[Tiab] OR hapale[Tiab] OR octodon[Tiab] OR chinchilla[Tiab] OR chinchillas[Tiab] OR gerbillinae[Tiab] OR gerbil[Tiab] OR gerbils[Tiab] OR jird[Tiab] OR jirds[Tiab] OR merione[Tiab] OR meriones[Tiab] OR rabbits[Tiab] OR rabbit[Tiab] OR hares[Tiab] OR hare[Tiab] OR diptera[Tiab] OR flies[Tiab] OR fly[Tiab] OR dipteral[Tiab] OR drosophila[Tiab] OR drosophilidae[Tiab] OR cats[Tiab] OR cat[Tiab] OR carus[Tiab] OR felis[Tiab] OR nematoda[Tiab] OR nematode[Tiab] OR nematodes[Tiab] OR sipunculida[Tiab] OR dogs[Tiab] OR dog[Tiab] OR canine[Tiab] OR canines[Tiab] OR canis[Tiab] OR sheep[Tiab] OR sheeps[Tiab] OR mouflon[Tiab] OR mouflons[Tiab] OR ovis[Tiab] OR goats[Tiab] OR goat[Tiab] OR capra[Tiab] OR capras[Tiab] OR rupicapra[Tiab] OR chamois[Tiab] OR haplorhini[Tiab] OR monkey[Tiab] OR monkeys[Tiab] OR anthropoidea[Tiab] OR anthropoids[Tiab] OR saguinus[Tiab] OR tamarin[Tiab] OR tamarins[Tiab] OR leontopithecus[Tiab] OR hominidae[Tiab] OR ape[Tiab] OR apes[Tiab] OR “pan paniscus”[Tiab] OR bonobo[Tiab] OR bonobos[Tiab] OR “pan troglodytes”[Tiab] OR gibbon[Tiab] OR gibbons[Tiab] OR siamang[Tiab] OR siamangs[Tiab] OR nomascus[Tiab] OR symphalangus[Tiab] OR chimpanzee[Tiab] OR chimpanzees[Tiab] OR prosimian[Tiab] OR prosimians[Tiab] OR “bush baby”[Tiab] OR bush babies[Tiab] OR galagos[Tiab] OR galago[Tiab] OR pongidae[Tiab] OR gorilla[Tiab] OR gorillas[Tiab] OR “pongo pygmaeus”[Tiab] OR orangutan[Tiab] OR orangutans[Tiab] OR lemur[Tiab] OR lemurs[Tiab] OR lemuridae[Tiab] OR horse[Tiab] OR horses[Tiab] OR equus[Tiab] OR cow[Tiab] OR calf[Tiab] OR bull[Tiab] OR chicken[Tiab] OR chickens[Tiab] OR gallus[Tiab] OR quail[Tiab] OR bird[Tiab] OR birds[Tiab] OR quails[Tiab] OR poultry[Tiab] OR poultries[Tiab] OR fowl[Tiab] OR fowls[Tiab]) NOT medline[sb]<br><br>OR<br><br>(reptile[Tiab] OR reptilia[Tiab] OR reptiles[Tiab] OR snakes[Tiab] OR snake[Tiab] OR lizard[Tiab] OR lizards[Tiab] OR alligator[Tiab] OR alligators[Tiab] OR crocodile[Tiab] OR crocodiles[Tiab] OR turtle[Tiab] OR turtles[Tiab] OR amphibian[Tiab] OR amphibians[Tiab] OR amphibia[Tiab] OR frog[Tiab] OR frogs[Tiab] OR bombina[Tiab] OR salientia[Tiab] OR toad[Tiab] OR toads[Tiab] OR “epidalea calamita”[Tiab] OR salamander[Tiab] OR salamanders[Tiab] OR eel[Tiab] OR eels[Tiab] OR fish[Tiab] OR fishes[Tiab] OR pisces[Tiab] OR catfish[Tiab] OR catfishes[Tiab] OR siluriformes[Tiab] OR arius[Tiab] OR heteropneustes[Tiab] OR sheatfish[Tiab] OR perch[Tiab] OR perches[Tiab] OR percidae[Tiab] OR perca[Tiab] OR trout[Tiab] OR trouts[Tiab] OR char[Tiab] OR chars[Tiab] OR salvelinus[Tiab] OR minnow[Tiab] OR cyprinidae[Tiab] OR carps[Tiab] OR carp[Tiab] OR zebrafish[Tiab] OR zebrafishes[Tiab] OR goldfish[Tiab] OR goldfishes[Tiab] OR guppy[Tiab] OR guppies[Tiab] OR chub[Tiab] OR chubs[Tiab] OR tinca[Tiab] OR barbels[Tiab] OR barbus[Tiab] OR pimephales[Tiab] OR promelas[Tiab] OR “poecilia reticulata”[Tiab] OR mullet[Tiab] OR mullets[Tiab] OR eel[Tiab] OR eels[Tiab] OR

	seahorse[Tiab] OR seahorses[Tiab] OR mugil curema[Tiab] OR atlantic cod[Tiab] OR shark[Tiab] OR sharks[Tiab] OR catshark[Tiab] OR anguilla[Tiab] OR salmonid[Tiab] OR salmonids[Tiab] OR whitefish[Tiab] OR whitefishes[Tiab] OR salmon[Tiab] OR salmons[Tiab] OR sole[Tiab] OR solea[Tiab] OR lamprey[Tiab] OR lampreys[Tiab] OR pumpkinseed[Tiab] OR sunfish[Tiab] OR sunfishes[Tiab] OR tilapia[Tiab] OR tilapias[Tiab] OR turbot[Tiab] OR turbots[Tiab] OR flatfish[Tiab] OR flatfishes[Tiab] OR sciuridae[Tiab] OR squirrel[Tiab] OR squirrels[Tiab] OR chipmunk[Tiab] OR chipmunks[Tiab] OR suslik[Tiab] OR susliks[Tiab] OR vole[Tiab] OR voles[Tiab] OR lemming[Tiab] OR lemmings[Tiab] OR muskrat[Tiab] OR muskrats[Tiab] OR lemmus[Tiab] OR otter[Tiab] OR otters[Tiab] OR marten[Tiab] OR martens[Tiab] OR martes[Tiab] OR weasel[Tiab] OR badger[Tiab] OR badgers[Tiab] OR ermine[Tiab] OR mink[Tiab] OR minks[Tiab] OR sable[Tiab] OR sables[Tiab] OR gulo[Tiab] OR gulos[Tiab] OR wolverine[Tiab] OR wolverines[Tiab] OR mustela[Tiab] OR llama[Tiab] OR llamas[Tiab] OR alpaca[Tiab] OR alpacas[Tiab] OR camelid[Tiab] OR camelids[Tiab] OR guanaco[Tiab] OR guanacos[Tiab] OR chiroptera[Tiab] OR chiropteras[Tiab] OR bat[Tiab] OR bats[Tiab] OR fox[Tiab] OR foxes[Tiab] OR iguana[Tiab] OR iguanas[Tiab] OR xenopus laevis[Tiab] OR parakeet[Tiab] OR parakeets[Tiab] OR parrot[Tiab] OR parrots[Tiab] OR donkey[Tiab] OR donkeys[Tiab] OR mule[Tiab] OR mules[Tiab] OR zebra[Tiab] OR zebras[Tiab] OR shrew[Tiab] OR shrews[Tiab] OR bison[Tiab] OR bisons[Tiab] OR buffalo[Tiab] OR buffaloes[Tiab] OR deer[Tiab] OR deers[Tiab] OR bear[Tiab] OR bears[Tiab] OR panda[Tiab] OR pandas[Tiab] OR “wild hog”[Tiab] OR “wild boar”[Tiab] OR fitchew[Tiab] OR fitch[Tiab] OR beaver[Tiab] OR beavers[Tiab] OR jerboa[Tiab] OR jerboas[Tiab] OR capybara[Tiab] OR capybaras[Tiab] OR canine [tiab] OR bovine [tiab] OR porcine [tiab] OR hog [tiab] OR hogs [tiab]) NOT medline[sb]
**EMBASE (via OVID)**	
Corticosteroids	exp steroid therapy/ or exp steroid/ or (adrenal cortex hormone or adrenal cortex hormones or adrenal cortical hormone or adrenal cortical hormones or adrenal cortical steroid or adrenal cortical steroids or adrenal steroid or adrenal steroids or adrenal steroid hormones or adreno cortical steroid or adreno cortical steroids or adreno corticosteroid or adreno corticosteroids or adrenocortical hormone or adrenocortical hormones or adrenocortical steroid or adrenocortical steroids or adrenocorticosteroid or adrenocorticosteroids or alclometasone or aldosterone or amcinonide or aristocort or aristospan or azmacort or beclomethasone or beclometasone or betamethasone or betametasone or benzodrocortisone or benzodrocortisones or budesonide or butixocort or celestone or chlormadinone acetate or chloroprednisone or ciclesonide or clobetasol or clobetasone or clocortolone or cloprednol or cloticasone or colocort or cortan or cortancyl or cortef or cortenema or cortexolone or cortical steroid or cortical steroids or cortico steroid or cortico steroids or corticoid or corticoids or corticosteroid or corticosteroids or corticosterone or corticotropin or cortifen or cortifoam or cortisol or cortisone or cortivazol or cortobenzolone or cortodoxone or cortone acetate or cortril or cotolone or cyproterone acetate or dacortin or decadron or

	decortin or decortisyl or deflazacort or dehydrocortisone or delta-cortisone or deltasone or deoxycorticosterone or deoxycortisol or deoxycortisone or deoxycortone or depomedrol or depo-medrol or dermocorticosteroid or dermocorticosteroids or desonide or desoximetasone or desoximethasone or desoxycorticosterone acetate or desoxycortone or dexametasone or dexamethasone or dexasone or dexpak or dichlorisone or diflorasone or difluocortolone or difluprednate or drocinonide phosphate or emflaza or encorton or encortone or entocort or epicortisol or etiprednol dicloacetate or fluclorolone or flucloronide or fludrocortisone acetate or fludrocortisone or fludroxycortide or flumetasone or flumethasone or flunisolide or fluocinolone acetonide or fluocinolone or fluocinonide or fluocortin or fluocortolone or fluoroformylone or fluorometolone or fluorometholone or flupamesone or fluperolone or fluprednidene or fluprednisolone or flurandrenolide or flurandrenolone or fluticasone or formocortal or glucocorticoid or glucocorticoids or halcinonide or halobetasol or halometasone or halopredone acetate or halopredone diacetate or hidex or hydrocort or hydrocortamate or hydrocortisone or hydroxycorticosteroid or hydroxycorticosteroids or hydroxypregnenolone or hydroxyprogesterone or icometasone enbutate or isoflupredone acetate or kenalog-10 or kenalog-40 or kenalog or ketoprogesterone or liquid pred or loteprednol or medrogestone or medrol or medroxyprogesterone acetate or medrysone or megestrol acetate or melengestrol or meprednisone or methapred or metapred or methylpred or methylprednisolone or metylprednisolone or meticorten or mometasone furoate or mometasone or nicocortonide acetate or nicocortonide or orapred or orasone or panafcort or panasol or paramethasone or parametasone or pediapred or prednazoline or prednicarbate or prednicot or prednidib or prednimustine or prednisol or prednisole or prednisolene or prednisollone or prednisolne or prednisoloe or prednisolole or prednisolon or prednisolona or prednisolonacetate or prednisolonate or prednisolone or prednisolonebisguanylhydrazone or prednisolonebisuccinate or prednisolonecarboxylate or prednisolonecarboxylates or prednisolones or prednisolonesodium or prednisolonester or prednisolonethe or prednisolono or prednisolonon or prednisolonum or prednisolum or prednisolut or prednisolute or prednisom or prednisome or prednison or prednisona or prednisone or prednisonlone or prednisonole or prednisonum or prednisonums or prednisoone or prednylidene or pregnenolone or prelone or progesterone or pronisone or rayos or rectodelt or rimexolone or rofleponide palmitate or segesterone or solu-cortef or sone or sterapred or steroids or ticabesone or timobesone acetate or tixocortol or tobradex or triamcinolone or trilostane or uceris or ulobetasol or ultracorten or winpred or zilretta).ti,ab,kw.
Lung	exp lung/ or exp lung development or (lung or lungs or alveol or alveola or alveolae or alveolaer or alveolair or alveolaire or alveolar or alveolararterial or alveolarcapillary or alveolarepithelial or alveolarepithelium or alveolargenesis or alveolarisation or alveolarization or alveolarized or alveolarterial or alveolary or alveolas or alveole or alveolear or alveoler or alveoles or alveoli or alveolia or alveoliar or alveolies or alveolis or alveolization or alveolli or alveolo or alveoloarterial or alveolobronchial or alveolobronchiolar or alveolocapillary or

	alveolocapillay or alveolocyte or alveolocytes or alveologenesis or alveoloid or alveolointerstial or alveolointerstitial or alveolopathies or alveolopathy or alveoloseptal or alveolous or alveolovascular or alveolo-vascular or alveolus or alveoluslike or airspace or airspaces or air-space or air-spaces or pulmonary).ti,ab,kw.
Animal	exp animal experiment/ or exp animal model/ or exp experimental animal/ or exp transgenic animal/ or exp male animal/ or exp female animal/ or exp juvenile animal/ OR animal/ OR chordata/ OR vertebrate/ OR tetrapod/ OR exp fish/ OR amniote/ OR exp amphibia/ OR mammal/ OR exp reptile/ OR exp sauropsid/ OR therian/OR exp monotremate/ OR placental mammals/ OR exp marsupial/ OR Euarchontoglires/ OR exp Afrotheria/ OR exp Boreoeutheria/ OR exp Laurasiatheria/ OR exp Xenarthra/ OR primate/ OR exp Dermoptera/ OR exp Glires/ OR exp Scandentia/ OR Haplorhini/ OR exp prosimian/ OR simian/ OR exp tarsiiform/ OR Catarrhini/ OR exp Platyrrhini/ OR ape/ OR exp Cercopithecidae/ OR hominid/ OR exp hylobatidae/ OR exp chimpanzee/ OR exp gorilla/ OR exp orang utan/ OR (animal OR animals OR pisces OR fish OR fishes OR catfish OR catfishes OR sheatfish OR silurus OR arius OR heteropneustes OR clarias OR gariepinus OR fathead minnow OR fathead minnows OR pimephales OR promelas OR cichlidae OR trout OR trouts OR char OR chars OR salvelinus OR salmo OR oncorhynchus OR guppy OR guppies OR millionfish OR poecilia OR goldfish OR goldfishes OR carassius OR auratus OR mullet OR mullets OR mugil OR curema OR shark OR sharks OR cod OR cods OR gadus OR morhua OR carp OR carps OR cyprinus OR carpio OR killifish OR eel OR eels OR anguilla OR zander OR sander OR lucioperca OR stizostedion OR turbot OR turbots OR psetta OR flatfish OR flatfishes OR plaice OR pleuronectes OR platessa OR tilapia OR tilapias OR oreochromis OR sarotherodon OR common sole OR dover sole OR solea OR zebrafish OR zebrafishes OR danio OR rerio OR seabass OR dicentrarchus OR labrax OR morone OR lamprey OR lampreys OR petromyzon OR pumpkinseed OR pumpkinseeds OR lepomis OR gibbosus OR herring OR clupea OR harengus OR amphibia OR amphibian OR amphibians OR anura OR salientia OR frog OR frogs OR rana OR toad OR toads OR bufo OR xenopus OR laevis OR bombina OR epidalea OR calamita OR salamander OR salamanders OR newt OR newts OR triturus OR reptilia OR reptile OR reptiles OR bearded dragon OR pogona OR vitticeps OR iguana OR iguanas OR lizard OR lizards OR anguis fragilis OR turtle OR turtles OR snakes OR snake OR aves OR bird OR birds OR quail OR quails OR coturnix OR bobwhite OR colinus OR virginianus OR poultry OR poultries OR fowl OR fowls OR chicken OR chickens OR gallus OR zebra finch OR taeniopygia OR guttata OR canary OR canaries OR serinus OR canaria OR parakeet OR parakeets OR grasskeet OR parrot OR parrots OR psittacine OR psittacines OR shelduck OR tadorna OR goose OR geese OR branta OR leucopsis OR woodlark OR lullula OR flycatcher OR ficedula OR hypoleuca OR dove OR doves OR geopelia OR cuneata OR duck OR ducks OR greylag OR graylag OR anser OR harrier OR circus pygargus OR red knot OR great knot OR calidris OR canutus OR godwit OR limosa OR lapponica OR meleagris OR gallopavo OR jackdaw OR corvus OR monedula OR ruff OR philomachus OR pugnax OR lapwing OR peewit OR plover OR vanellus OR swan OR

	cygnus OR columbianus OR bewickii OR gull OR chroicocephalus OR ridibundus OR albifrons OR great tit OR parus OR aythya OR fuligula OR streptopelia OR risoria OR spoonbill OR platalea OR leucorodia OR blackbird OR turdus OR merula OR blue tit OR cyanistes OR pigeon OR pigeons OR columba OR pintail OR anas OR starling OR sturnus OR owl OR athene noctua OR pochard OR ferina OR cockatiel OR nymphicus OR hollandicus OR skylark OR alauda OR tern OR sterna OR teal OR crecca OR oystercatcher OR haematopus OR ostralegus OR shrew OR shrews OR sorex OR araneus OR crocidura OR russula OR european mole OR talpa OR chiroptera OR bat OR bats OR eptesicus OR serotinus OR myotis OR dasycneme OR daubentonii OR pipistrelle OR pipistrellus OR cat OR cats OR felis OR catus OR feline OR dog OR dogs OR canis OR canine OR canines OR otter OR otters OR lutra OR badger OR badgers OR meles OR fitchew OR fitch OR foumart or foulmart OR ferrets OR ferret OR polecat OR polecats OR mustela OR putorius OR weasel OR weasels OR fox OR foxes OR vulpes OR common seal OR phoca OR vitulina OR grey seal OR halichoerus OR horse OR horses OR equus OR equine OR equidae OR donkey OR donkeys OR mule OR mules OR pig OR pigs OR swine OR swines OR hog OR hogs OR boar OR boars OR porcine OR piglet OR piglets OR sus OR scrofa OR llama OR llamas OR lama OR glama OR deer OR deers OR cervus OR elaphus OR cow OR cows OR bos taurus OR bos indicus OR bovine OR bull OR bulls OR cattle OR bison OR bisons OR sheep OR sheeps OR ovis aries OR ovine OR lamb OR lambs OR mouflon OR mouflons OR goat OR goats OR capra OR caprine OR chamois OR rupicapra OR leporidae OR lagomorpha OR lagomorph OR rabbit OR rabbits OR oryctolagus OR cuniculus OR laprine OR hares OR lepus OR rodentia OR rodent OR rodents OR murinae OR mouse OR mice OR mus OR musculus OR murine OR woodmouse OR apodemus OR rat OR rats OR rattus OR norvegicus OR guinea pig OR guinea pigs OR cavia OR porcellus OR hamster OR hamsters OR mesocricetus OR cricetulus OR cricetus OR gerbil OR gerbils OR jird OR jirds OR meriones OR unguiculatus OR jerboa OR jerboas OR jaculus OR chinchilla OR chinchillas OR beaver OR beavers OR castor fiber OR castor canadensis OR sciuridae OR squirrel OR squirrels OR sciurus OR chipmunk OR chipmunks OR marmot OR marmots OR marmota OR suslik OR susliks OR spermophilus OR cynomys OR cottonrat OR cottonrats OR sigmodon OR vole OR voles OR microtus OR myodes OR glareolus OR primate OR primates OR prosimian OR prosimians OR lemur OR lemurs OR lemuridae OR loris OR bush baby OR bush babies OR bushbaby OR bushbabies OR galago OR galagos OR anthropoidea OR anthropoids OR simian OR simians OR monkey OR monkeys OR marmoset OR marmosets OR callithrix OR cebuella OR tamarin OR tamarins OR saguinus OR leontopithecus OR squirrel monkey OR squirrel monkeys OR saimiri OR night monkey OR night monkeys OR owl monkey OR owl monkeys OR douroucoulis OR aotus OR spider monkey OR spider monkeys OR ateles OR baboon OR baboons OR papio OR rhesus monkey OR macaque OR macaca OR mulatta OR cynomolgus OR fascicularis OR green monkey OR green monkeys OR chlorocebus OR vervet OR vervets OR pygerythrus OR hominoidea OR ape OR apes OR hylobatidae OR gibbon OR gibbons OR siamang OR siamangs OR nomascus OR symphalangus OR hominidae OR orangutan OR orangutans OR

	pongo OR chimpanzee OR chimpanzees OR pan troglodytes OR bonobo OR bonobos OR pan paniscus OR gorilla OR gorillas OR troglodytes).ti,ab,kw.
Newborn animal	exp newborn/ or exp baby/or exp prematurity/ or exp juvenile animal/ or (infant or babies or baby or immature or immaturity or infant or infants or juvenile or juveniles or neonate or neonates or newborn or newborns or premature or prematurely or prematurity or preterm or pre-term or litter or litters or litter size or after birth or postnatal or postnatally or post-natal or post-natally or developmental or calf or calfs or chulengos or colt or colts or cosset or cub or cubs or farrow or farrows or fawn or fawns or fillies or filly or foal or foals or hoglet or hoglets or joey or joeys or kid or kids or kitten or kittens or lamb or lambkin or lambs or leveret or leverets or nestling or nestlings or piglet or piglets or puggles or pup or puppies or puppy or pups or shoat or shoats or tumbler or tumblers or suckling animal or suckling animals or whelp or whelps).ti,ab,kw.
Newborn animal	Infant, Newborn[MeSH] OR Infant[tiab] OR Babies[tiab] OR Baby[tiab] OR Immature[tiab] OR Immaturity[tiab] OR Infant[tiab] OR Infants[tiab] OR Juvenile[tiab] OR Juveniles[tiab] OR Neonate[tiab] OR Neonates[tiab] OR Newborn[tiab] OR Newborns[tiab] OR Premature[tiab] OR Prematurity[tiab] OR Preterm[tiab] OR pre-term[tiab] OR Litter[tiab] OR Litters[tiab] OR "Litter size"[tiab] OR "Premature Birth"[Mesh] OR “after birth”[tiab] OR Prematurely[tiab] OR Postnatal[tiab] OR Postnatally[tiab] OR post-natal[tiab] OR post-natally[tiab] OR Developmental[tiab] OR "Animals, Suckling"[Mesh] OR Animals, Newborn[MeSH] OR Calf[tiab] OR Calfs[tiab] OR Chulengos[tiab] OR Colt[tiab] OR Colts[tiab] OR Cosset[tiab] OR Cub[tiab] OR Cubs[tiab] OR Farrow[tiab] OR Farrows[tiab] OR Fawn[tiab] OR Fawns[tiab] OR Fillies[tiab] OR Filly[tiab] OR Foal[tiab] OR Foals[tiab] OR Hoglet[tiab] OR Hoglets[tiab] OR Joey[tiab] OR Joeys[tiab] OR Kid[tiab] OR Kids[tiab] OR Kitten[tiab] OR Kittens[tiab] OR Lamb[tiab] OR Lambkin[tiab] OR Lambs[tiab] OR Leveret[tiab] OR Leverets[tiab] OR Nestling[tiab] OR Nestlings[tiab] OR Piglet[tiab] OR Piglets[tiab] OR Puggles[tiab] OR Pup[tiab] OR Puppies[tiab] OR Puppy[tiab] OR Pups[tiab] OR Shoat[tiab] OR Shoats[tiab] OR Tumbler[tiab] OR Tumblers[tiab] OR Whelp[tiab] OR Whelps[tiab]

Supplement 3. Flow diagram of study selection according to PRISMA 2020 statement.[19]

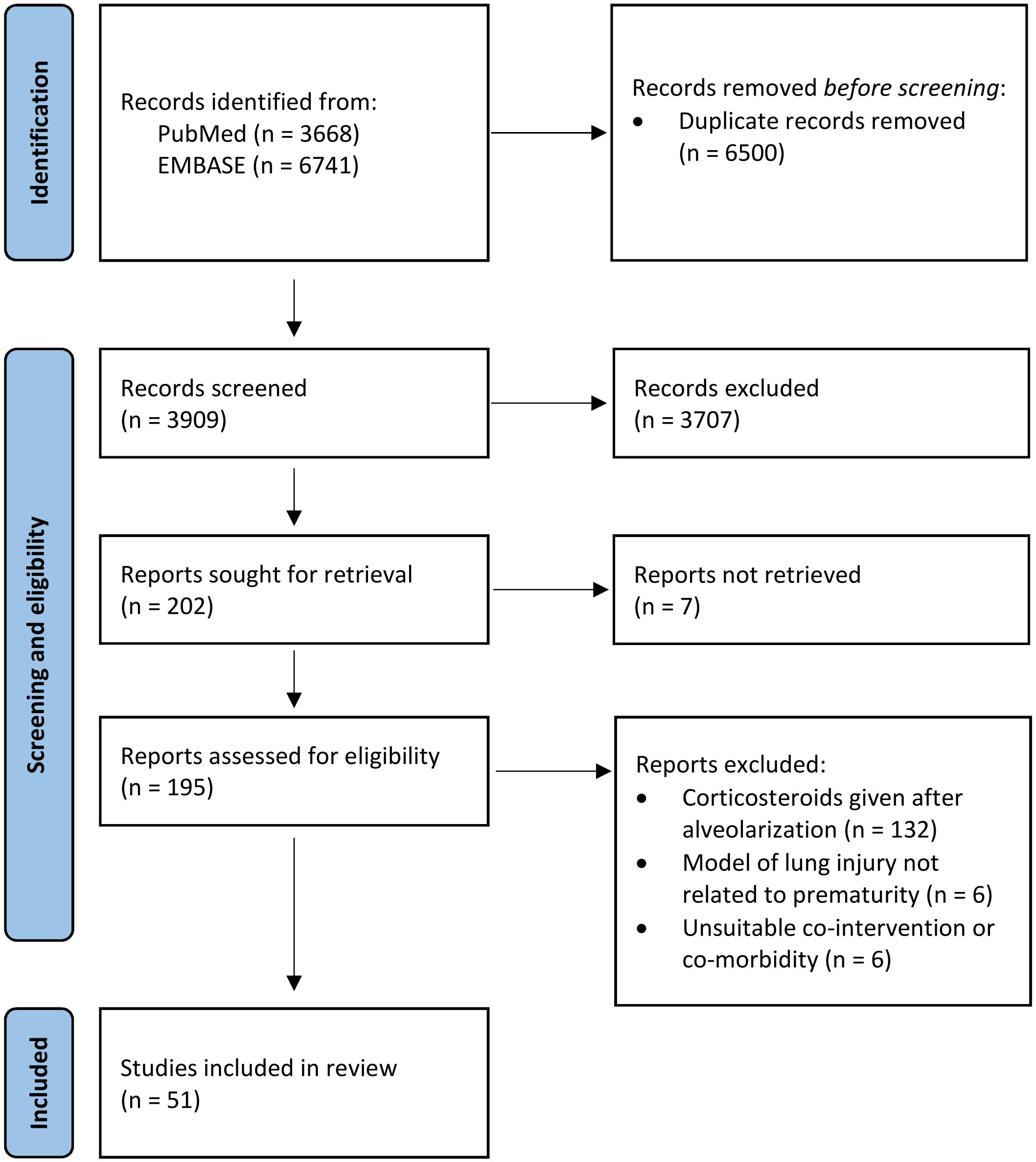

Supplement 4. Descriptive mortality.

**Study**	Species	Lung injury model or co-intervention	Type of GC	Timing of GC treatment (PND)	PND at measurement of mortality	In- or decrease in mortality†
Dallas[28]	Rat	Normoxia	Dexa	0-6<br>0-9<br>0-13	7<br>10<br>14	+41%*<br>+66%*<br>+79%*
Dallas[28]	Rat	Hyperoxia	Dexa	0-6<br>0-9<br>0-13	7<br>10<br>14	+15%<br>-18%<br>-5%
Dallas[28]	Rat	Normoxia	Dexa	0-6<br>0-9<br>0-13	7<br>10<br>14	+13%<br>+13%<br>+16%
Dallas[28]	Rat	Hyperoxia	Dexa	0-6<br>0-9<br>0-13	7<br>10<br>14	-14%<br>-39%<br>-41%*
Dallas[28]	Rat	Normoxia	Dexa	0-6<br>0-9<br>0-13	7<br>10<br>14	+3%<br>+28%<br>+29%
Dallas[26]	Rat	Hyperoxia	Dexa	0-6<br>0-9<br>0-13	7<br>10<br>14	-21%<br>-66%*<br>-77%*
Dallas[28]	Rat	Normoxia	Dexa	0-6<br>0-9<br>0-13	7<br>10<br>14	-9%<br>+3%<br>+4%

Dallas[28]	Rat	Hyperoxia	Dexa	0-6<br>0-9<br>0-13	7<br>10<br>14	-21%<br>-61%*<br>-70%*
Dallas[28]	Rat	Normoxia	Dexa	4-6	7	+5%
Dallas[28]	Rat	Hyperoxia	Dexa	4-6	7	-5%
Fayon[29]	Rat		Dexa	4-13	14	No mortality
Fayon[29]	Rat		HCS	4-13	14	No mortality
Hu[33]	Rat	Hyperoxia	Dexa	NR	13	No mortality
Ishikawa[34]	Rat	Bleomycin	Dexa	0-13	14	0%
Kim[35]	Rat	Prenatal BMS, Hyperoxia	Dexa	5-7	14	0%
Kim[35]	Rat	Hyperoxia	Dexa	5-7	14	-21%
Lee[37]	Rat	Prenatal LPS, Hyperoxia	Dexa	1-6	14	+10%*
Lee[37]	Rat	Prenatal LPS, Hyperoxia	HCS	1-6	14	+2%
Massaro[42]	Rat		Dexa	1-6	7	No mortality
Ohtsu[71]	Mouse	Normoxia	Dexa 1 mg/kg	14-17	18	No mortality
Ohtsu[71]	Mouse	Hyperoxia	Dexa 1 mg/kg	14-17	18	No mortality
Ohtsu[71]	Mouse	Normoxia	Dexa 5 mg/kg	14-17	18	+13%
Ohtsu[71]	Mouse	Hyperoxia	Dexa 5 mg/kg	14-17	18	+13%
Özer Bekmez[47]	Rat	Hyperoxia	Dexa	15-21	22	-13%
Özer Bekmez[47]	Rat	Hyperoxia	HCS	15-21	22	-13%
Özer Bekmez[47]	Rat	Hyperoxia	MPS	15-21	22	-13%
Sahebjemi[51]	Rat		Dexa	4-13	99	+5%

Thibeault[56]	Rat	Normoxia	Dexa	0-7	60	+16%
Thibeault[56]	Rat	Hyperoxia	Dexa	0-7	60	+43%
Town[74]	Guinea pig	Prematurity, Normoxia	Dexa	3-5	7	+4%
Town[74]	Guinea pig	Prematurity, Hyperoxia	Dexa	3-5	7	+6%
Valencia[60]	Rat		Dexa 0.1 mg/kg	5-7	45	No mortality
Valencia[60]	Rat		Dexa 0.5 mg/kg	5-7	45	No mortality
Veness-Meehan[61]	Rat	Hyperoxia	Dexa	3-13	14	-6%
Veness-Meehan[61]	Rat	Hyperoxia, RA	Dexa	3-13	14	-20%

GC: glucocorticoid, PND: postnatal day, †: absolute difference in mortality (%) in newborn animals treated with corticosteroids compared to newborn animals exposed to the same conditions but without corticosteroids, BMS: betamethasone, LPS: lipopolysaccharide, RA: retinoic acid, Dexa: dexamethasone, HCS: hydrocortisone, MPS: methylprednisolone, No mortality: no animals died during the experiments, Mortality 0%: mortality was equal in group with corticosteroids and control group. *: significant difference.

Supplement 5. Forest plot body weight (growth).

Meta-analysis of the outcome growth, subgrouped by age at evaluation

Study or Subgroup	Corticosteroid Mean	Corticosteroid SD	Corticosteroid Total	Control Mean	Control SD	Control Total	Weight	Std. Mean Difference IV, Random, 95% CI
**<PND15**								
Bhatt 2000-PND10	0.09	0.6	24	1.26	0.5	24	1.4%	-2.08 [-2.80, -1.37]
Blanco 1989-PND14	24.9	0.7	3	29.1	1.4	3	0.7%	-3.04 [-6.38, 0.30]
Fayon 2002-PND15-DEX	24.3	1.4	12	28.1	2.7	6	1.3%	-1.90 [-3.11, -0.69]
Fayon 2002-PND15-HCS	28.8	1.4	12	28.1	2.7	6	1.3%	0.35 [-0.64, 1.34]
Garber 2006-PND05-CSO	11.4	0.6	8	12.9	1.1	8	1.3%	-1.60 [-2.77, -0.43]
Garber 2006-PND05-RA	11.7	0.5	7	12.9	0.8	8	1.3%	-1.67 [-2.89, -0.44]
Garber 2006-PND10-CSO	15	0.6	8	17.1	1.1	8	1.2%	-2.24 [-3.57, -0.91]
Garber 2006-PND10-RA	15.4	0.8	7	16.8	1.1	8	1.3%	-1.35 [-2.51, -0.19]
Garber 2006-PND15-CSO	5.8	2	8	31.4	2.3	8	0.4%	-11.23 [-15.82, -6.64]
Garber 2006-PND15-RA	26	2.4	7	28.8	2.3	8	1.3%	-1.12 [-2.24, -0.01]
Gesche 2011-PND03-CON	0	0.4	19	2.3	0.2	27	1.1%	-7.57 [-9.30, -5.85]
Gesche 2011-PND03-CON-F	6.2	0.2	10	8.5	0.3	10	0.7%	-8.64 [-11.75, -5.53]
Gesche 2011-PND03-CON-M	7	0.4	11	9.2	0.2	7	0.9%	-6.18 [-8.65, -3.71]
Gesche 2011-PND03-rhKGF	0.2	0.4	19	1.4	0.3	19	1.3%	-3.32 [-4.34, -2.31]
Gesche 2011-PND03-rhKGF-F	6.9	0.4	11	8.2	0.3	5	1.1%	-3.28 [-4.97, -1.60]
Gesche 2011-PND03-rhKGF-M	6.9	0.4	7	8	0.3	12	1.2%	-3.10 [-4.55, -1.65]
Gesche 2011-PND07-CON-F	12.2	0.5	15	15	0.7	11	1.2%	-4.58 [-6.14, -3.02]
Gesche 2011-PND07-CON-M	11.7	0.4	6	14.6	0.7	11	1.0%	-4.47 [-6.45, -2.48]
Gesche 2011-PND07-rhKGF	0.5	0.3	19	3.2	0.2	19	0.9%	-10.37 [-12.91, -7.83]
Gesche 2011-PND07-rhKGF-F	12.5	0.5	8	15.2	0.7	13	1.2%	-4.09 [-5.72, -2.46]
Gesche 2011-PND07-rhKGF-M	11.6	0.4	16	14.3	0.4	11	1.0%	-6.55 [-8.58, -4.51]
Gesche 2011-PND15-CON	0.1	0.5	19	4	0.3	21	0.9%	-9.39 [-11.64, -7.13]
Gesche 2011-PND15-rhKGF	2.4	0.5	19	3.6	0.2	19	1.4%	-3.09 [-4.06, -2.12]
Ishikawa 2022-BLEO	18.98	5.39	5	25.92	3.62	5	1.2%	-1.37 [-2.82, 0.09]
Kim 2019-PND08-BMS-HYP	12.4	1	14	16.2	1.4	16	1.3%	-3.00 [-4.09, -1.92]
Kim 2019-PND08-VEH-HYP	12	1.1	11	16.9	0.9	11	1.1%	-4.69 [-6.43, -2.95]
Kim 2019-PND14-BMS-HYP	23.1	1.7	11	26	2.1	11	1.4%	-1.46 [-2.42, -0.50]
Kim 2019-PND14-VEH-HYP	23.1	1.6	12	25.8	1.4	10	1.3%	-1.72 [-2.73, -0.71]
Lee 2012-PND07-DEX-HYP	11.9	1.7	14	16.8	2.7	9	1.3%	-2.21 [-3.30, -1.12]
Lee 2012-PND07-HCS-HYP	16.7	0.7	12	16.8	2.7	9	1.4%	-0.05 [-0.92, 0.81]
Lee 2012-PND14-DEX-HYP	27	4.1	14	32.7	5.6	9	1.4%	-1.16 [-2.08, -0.25]
Lee 2012-PND14-HCS-HYP	29	4.9	12	32.7	5.6	9	1.4%	-0.68 [-1.58, 0.21]
Massaro 1985-PND04-0,1-DNA	8.9	1.6	10	9.2	1.8	2	1.2%	-0.17 [-1.69, 1.35]
Massaro 1985-PND14-0,05	28	1.9	7	28.7	2.2	2	1.2%	-0.32 [-1.90, 1.26]
Massaro 1985-PND14-0,1	25.5	1.1	7	28.7	2.2	2	1.0%	-2.16 [-4.22, -0.10]
Massaro 1985-PND14-0,2	24.3	1	4	28.7	2.2	2	0.7%	-2.51 [-5.48, 0.45]
Massaro 1985-PND14-0,3	19.2	1.2	3	28.7	2.2	2	0.3%	-4.31 [-10.37, 1.76]
Massaro 1985-PND14-0,4	20.2	1.2	4	28.7	2.2	2	0.4%	-4.49 [-9.15, 0.17]
Massaro 1985-PND14-0,5	14.2	2.6	3	28.7	2.2	2	0.3%	-4.26 [-10.27, 1.75]
Massaro 1985-PND28-0,1	83.3	5.2	3	91	6.9	2	1.0%	-0.96 [-3.17, 1.25]
Massaro 1985-PND28-0,1-DNA	80.3	10.8	10	81.1	7.5	2	1.2%	-0.07 [-1.59, 1.45]
Massaro 1985-PND28-0,5	41	3.5	3	91	6.9	2	0.1%	-7.42 [-17.56, 2.73]
Massaro 1986-1-PND07	11.8	1.4	3	14.1	0.7	3	0.9%	-1.66 [-3.93, 0.60]
Massaro 1986-1-PND07-DNA	13.5	0.9	5	14.3	0.7	5	1.2%	-0.90 [-2.23, 0.44]
Massaro 1986-2-PND06	12	1.2	6	13	0.7	5	1.3%	-0.91 [-2.18, 0.37]
Massaro 1986-2-PND14	26.2	1.3	10	28.9	2.1	17	1.4%	-1.41 [-2.29, -0.53]
Massaro 1996-PND14-CON	24.8	2.2	5	28.9	2.5	5	1.2%	-1.57 [-3.10, -0.05]
Massaro 1996-PND14-RA	23.9	1.2	6	29.4	2.7	6	1.1%	-2.43 [-4.07, -0.79]
Ross 2007-CON	4.5	0.2	4	4.6	0.4	4	1.2%	-0.27 [-1.67, 1.12]
Ross 2007-RA	3	0.6	4	4.5	0.2	4	0.9%	-2.92 [-5.35, -0.48]
Srinivasan 2002-CON	106	10	5	104	6	5	1.3%	0.22 [-1.03, 1.46]
Srinivasan 2002-CON-FUNCTION	117	27	13	128	24	10	1.4%	-0.41 [-1.25, 0.42]
Srinivasan 2002-RA	96	7	5	105	6	5	1.2%	-1.25 [-2.67, 0.18]
Srinivasan 2002-RA-FUNCTION	118	0	14	134	23	9		Not estimable
Tschanz 1995-PND04	10.8	0.2	4	10.8	0.6	4	1.2%	0.00 [-1.39, 1.39]
Tschanz 1995-PND07	10.9	0.8	4	13.3	1.8	4	1.1%	-1.50 [-3.23, 0.23]
Tschanz 1995-PND10	21	1.2	4	22.5	0.8	4	1.1%	-1.28 [-2.92, 0.36]
Tschanz 1995-PND13	25	1	4	26.5	0.4	4	1.1%	-1.71 [-3.53, 0.11]
Tschanz 2003-PND04	12.6	0.9	5	13.1	0.9	5	1.3%	-0.50 [-1.77, 0.77]
Tschanz 2003-PND10	24.5	0.9	5	27	3.8	5	1.3%	-0.82 [-2.14, 0.50]
**Subtotal (95% CI)**			**540**			**485**	**65.0%**	**-2.35 [-2.82, -1.87]**
Heterogeneity: $Tau^2 = 2.60$; $Chi^2 = 370.58$, df = 58 (P < 0.00001); $I^2 = 84\%$								
Test for overall effect: Z = 9.71 (P < 0.00001)								
**≥PND15**								
Blanco 1989-PND60	331	13	3	326	2.7	3	1.1%	0.43 [-1.23, 2.08]
Garber 2006-PND30-CSO	126	17	8	164	19.8	8	1.3%	-1.95 [-3.20, -0.70]
Garber 2006-PND30-RA	125	26.5	7	150	22.6	8	1.3%	-0.96 [-2.05, 0.13]
Gesche 2011-PND21-CON	4	0.8	6	5.7	0.8	8	1.2%	-1.99 [-3.36, -0.62]
Gesche 2011-PND21-rhKGF	3.9	0.8	6	4.3	0.6	6	1.3%	-0.52 [-1.68, 0.64]
Kamei 2014-PND42	37.8	8.8	3	28	3.4	3	1.0%	1.18 [-0.79, 3.14]
Ozer-Bekmez 2022-PND22-DEX	26.79	8.46	13	30.55	5.01	4	1.3%	-0.45 [-1.59, 0.68]
Ozer-Bekmez 2022-PND22-HCS	26.63	5.01	13	30.55	5.01	4	1.3%	-0.74 [-1.90, 0.41]
Ozer-Bekmez 2022-PND22-MPS	29.92	4.07	13	30.55	5.01	4	1.3%	-0.14 [-1.26, 0.98]
Sahebjemi 1989-PND32	119.4	5.8	20	121.4	10.3	20	1.4%	-0.23 [-0.86, 0.39]
Sahebjemi 1989-PND62	336.5	17.9	20	330.1	22.7	19	1.4%	0.31 [-0.32, 0.94]
Sahebjemi 1989-PND92	445.2	28.6	20	439.7	30.9	19	1.4%	0.18 [-0.45, 0.81]
Sahebjemi 1989-PND99-LUNG	508	22	6	486	31.3	5	1.3%	0.76 [-0.49, 2.01]
Sahebjemi 1989-PND99-WEIGHT	503.7	40.7	20	494.7	26.6	19	1.4%	0.26 [-0.38, 0.89]
Stinchcombe 2008-ICR-CON	34.4	5	13	39.3	4.2	12	1.4%	-1.02 [-1.87, -0.18]
Stinchcombe 2008-NIHS-CON	30.5	2.8	8	30.6	2	11	1.4%	-0.04 [-0.95, 0.87]
Theogaraj 2006	244	11.2	5	234	8.9	5	1.2%	0.89 [-0.44, 2.23]
Thibeault 1993-HYP	246	25	6	266	29	6	1.3%	-0.68 [-1.86, 0.50]
Thibeault 1993-NORM	239	18	9	281	33	7	1.3%	-1.56 [-2.72, -0.39]
Tschanz 1995-PND21	46.6	2.8	4	47.5	2.2	4	1.2%	-0.31 [-1.71, 1.09]
Tschanz 1995-PND36	139	10	4	135	10	4	1.2%	0.35 [-1.06, 1.75]
Tschanz 1995-PND44	216	4	4	211	14	4	1.2%	0.42 [-0.99, 1.84]
Tschanz 1995-PND60	328	12	4	346	22	4	1.2%	-0.88 [-2.40, 0.63]
Tschanz 2003-PND21	69.3	1.8	5	79.2	4.5	5	1.1%	-2.61 [-4.53, -0.69]
Tschanz 2003-PND36	226	8.3	5	209	8.9	5	1.2%	1.78 [0.19, 3.38]
Tschanz 2003-PND60	372	20.6	5	418	24.8	5	1.2%	-1.82 [-3.43, -0.21]
Valencia 2003-PND21-HIGH	37.8	1.3	10	45.5	0.9	5	0.8%	-6.08 [-8.84, -3.33]
Valencia 2003-PND21-LOW	39.3	1.9	10	45.5	0.9	5	1.1%	-3.52 [-5.34, -1.70]
**Subtotal (95% CI)**			**250**			**212**	**35.0%**	**-0.51 [-0.90, -0.12]**
Heterogeneity: $Tau^2 = 0.68$; $Chi^2 = 87.20$, df = 27 (P < 0.00001); $I^2 = 69\%$								
Test for overall effect: Z = 2.58 (P = 0.010)								
**Total (95% CI)**			**790**			**697**	**100.0%**	**-1.72 [-2.08, -1.35]**
Heterogeneity: $Tau^2 = 2.28$; $Chi^2 = 572.72$, df = 86 (P < 0.00001); $I^2 = 85\%$								
Test for overall effect: Z = 9.27 (P < 0.00001)								
Test for subgroup differences: $Chi^2 = 34.60$, df = 1 (P < 0.00001), $I^2 = 97.1\%$								

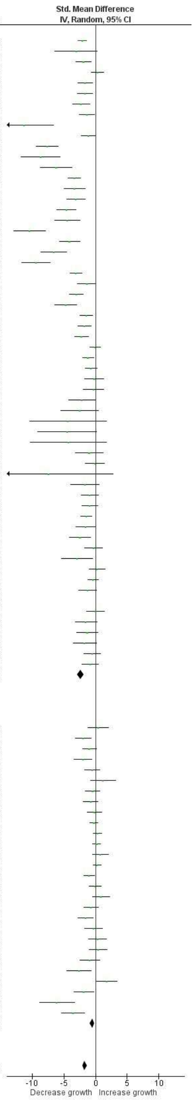

Supplement 5 Forest plot of meta-analysis comparing the effect of corticosteroids on body weight for the subgroup age at evaluation of outcome. Effect size is calculated as Standardized Mean Difference (SMD), with a 95% confidence interval (95% CI) in a random effects model. PND: postnatal day, DEX: dexamethasone, HCS: hydrocortisone, CSO: cottonseed oil, RA: retinoic acid, CON: control, F: female, M: male, rhKGF: recombinant human keratinocyte growth factor, BLEO: bleomycin, BMS: betamethasone, HYP: hyperoxia, VEH: vehicle, MPS: methylprednisolone, NORM: normoxia. DNA, FUNCTION, LUNG, WEIGHT, HIGH, and LOW: different experimental protocols. ICR and NIH: different mouse strains.
